# Simple direct formation of self-assembled N-heterocyclic carbene monolayers on gold and their application in biosensing

**DOI:** 10.1038/ncomms12654

**Published:** 2016-09-02

**Authors:** Cathleen M. Crudden, J. Hugh Horton, Mina R. Narouz, Zhijun Li, Christene A. Smith, Kim Munro, Christopher J. Baddeley, Christian R. Larrea, Benedict Drevniok, Bheeshmon Thanabalasingam, Alastair B. McLean, Olena V. Zenkina, Iraklii I. Ebralidze, Zhe She, Heinz-Bernhard Kraatz, Nicholas J. Mosey, Lisa N. Saunders, Akiko Yagi

**Affiliations:** 1 Department of Chemistry, Queen's University, 90 Bader Lane, Kingston, Ontario, Canada K7L 3N6; 2 Institute of Transformative Bio-Molecules (WPI-ITbM), Nagoya University, Chikusa, Nagoya 464-8602, Japan; 3 Protein Function Discovery Facility, Queen's University, Kingston, Ontario, Canada K7L 3N6; 4 EaStCHEM School of Chemistry, University of St Andrews, St Andrews, Fife KY16 9ST, UK; 5 Department of Physics, Engineering Physics and Astronomy, Queen's University, Kingston, Ontario, Canada K7L 3N6; 6 Department of Physical and Environmental Sciences, University of Toronto Scarborough, Toronto, Ontario, Canada M1C 1A4; 7 Department of Chemistry, University of Toronto, Toronto, Ontario, Canada M5S 3H6

## Abstract

The formation of organic films on gold employing N-heterocyclic carbenes (NHCs) has been previously shown to be a useful strategy for generating stable organic films. However, NHCs or NHC precursors typically require inert atmosphere and harsh conditions for their generation and use. Herein we describe the use of benzimidazolium hydrogen carbonates as bench stable solid precursors for the preparation of NHC films in solution or by vapour-phase deposition from the solid state. The ability to prepare these films by vapour-phase deposition permitted the analysis of the films by a variety of surface science techniques, resulting in the first measurement of NHC desorption energy (158±10 kJ mol^−1^) and confirmation that the NHC sits upright on the surface. The use of these films in surface plasmon resonance-type biosensing is described, where they provide specific advantages versus traditional thiol-based films.

## Abstract (web summary)

Self-assembled monolayers (SAMs) have shown tremendous number of applications but can suffer from low stability. Here, the authors report air and bench stable carbene precursors allowing facile SAM formation, and furthermore demonstrate an application in biosensing

## Graphical abstract

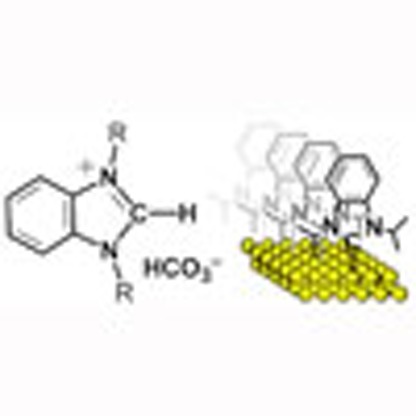

The use of self-assembled monolayers (SAMs) on gold^1^,^2^,^3^ as an interface between metal surfaces and organic species has had a remarkable impact on molecular electronics, surface patterning and biosensing^2^,^4^. Thiol-based thin-film-on-metal biosensors employing surface plasmon resonance (SPR) effects are routinely used for the detection of nanogram quantities of biomolecules in real time without the need for labelling^5^,^6^; however, challenges with film stability lead to low shelf life, poor bilayer film quality and increased nonspecific adsorption. Despite these important applications, thiol-based SAMs suffer from degradation in air, thermal instability and sensitivity to oxidation. Although the use of bidentate or polymeric thiols^7^,^8^ are important approaches to improve thiol SAMs, recent reports from Weidner *et al.*^9^, Johnson and colleagues^10^, and Crudden *et al.*^11^ have documented the intriguing possibility of employing N-heterocyclic carbenes (NHCs) as alternatives to thiols in the preparation of SAMs on metal surfaces^12^.

These NHC-based films display unprecedented chemical, pH, oxidative and electrochemical stability, making them viable alternatives to thiol-based films on gold^11^. However, free NHCs are typically prepared under highly specialized conditions, by deprotonation of the corresponding imidazolium salts with strong bases such as KO*t*Bu, NaH and KHMDS, a process that must be carried out under inert atmosphere, with rigorous exclusion of air and moisture (Fig. 1a)^13^,^14^,^15^. Depending on the base, contamination of the metal surface has been shown to be problematic^10^. As an alternative, we explored single-source NHC precursors that would not require strong base for their preparation or during deposition. In particular, our attention was drawn to hydrogen carbonate salts^16^, in which the counteranion also serves as the base to deprotonate the imidazolium cation. Carbon dioxide and water are the only byproducts^17^,^18^. Previous work from Johnson and colleagues^10^ includes the use of imidazolium carboxylates, which are also highly valuable for the preparation of NHC films from stable precursors. Film formation from this precursor was followed using quartz crystal microbalance techniques, which also enabled monitoring of chemical modifications to the NHC film. However, the preparation of these reagents typically requires strong base and inert atmosphere or reagents with high toxicity; therefore, we sought out a simple, scalable and user-friendly method that would permit the preparation of alternative NHC precursors.

Herein we report the development of air- and bench-stable single-source precursors and straightforward deposition methods that will enable researchers to examine these films in a wide range of applications. In addition, we quantify key bonding characteristics and describe the first example of the use of NHC films in functional devices, namely in SPR-based biosensing. These films provide opportunities for sensing under conditions previously not optimal using state-of-the-art thiol films, for example, in extremes of pH.

## Results

### NHC hydrogen carbonate synthesis and film generation

**Figure 1:**
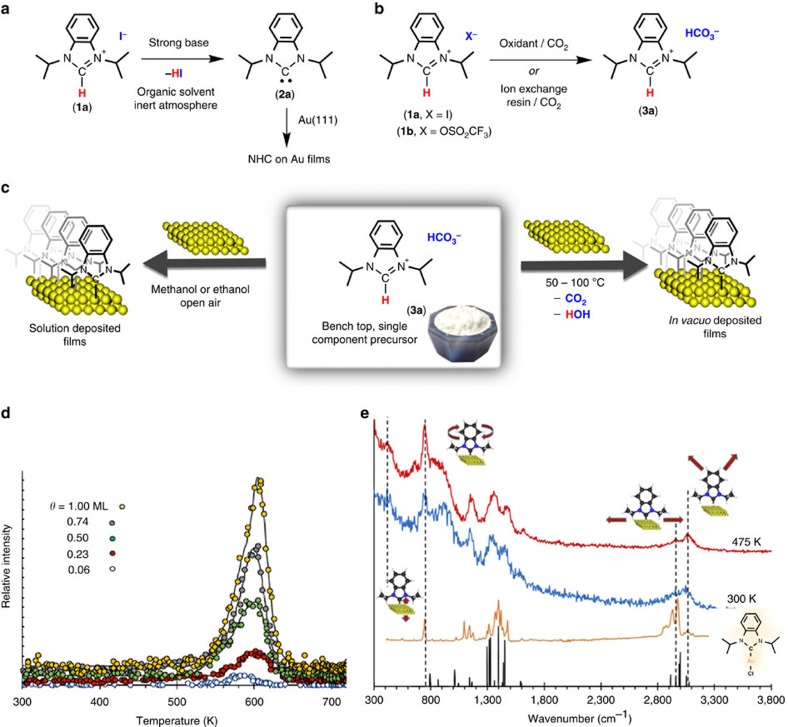
Single component easily handled NHC precursors and films. (**a**) Strong base method typically employed for the generation of NHCs. (**b**) Synthesis of benzimidazolium hydrogen carbonate (**3a**). (**c**) Preparation of NHC films from **3a** in organic solvent at 293 K or by heating the neat solid *in vacuo*. (**d**) TPD of signal at *m*/*z*=41 showing a maximum desorption temperature of 605 K. Θ (Theta)=1 monolayer (ML). (**e**) HREELS study of **3a** on Au(111) at 300 K (blue), after annealing to 475 K and cooling to 300 K (red), solution spectra for molecular analog NHC-Au-Cl (orange) and a simulation showing the calculated vibrational modes, which have dipole components normal to the surface (black).

Seeking to develop alternative methods to the use of strong base for the generation of free carbenes through deprotonation (**1a** to **2a**, Fig. 1a), we explored literature procedures for the preparation of imidazolium hydrogen carbonates via salt metathesis between imidazolium iodide salts and KHCO_3_ (ref. 16). In our hands, this procedure gave variable levels of exchange of iodide for hydrogen carbonate. The high affinity of gold for iodide then posed a problem in the creation of clean NHC films. Thus, two new synthetic procedures were developed (Fig. 1b). The first involved oxidative removal of the iodide counterion in the presence of hydrogen peroxide and CO_2_, and the second employed an ion exchange resin^19^. Once pure, diisopropyl-benzimidazolium hydrogen carbonate (**3a**) proved to be highly effective for the formation of high-quality NHC films either in solution or in the gas phase, directly from the solid (Fig. 1c). If films with no trace of iodide were required, the trifluoromethylsulfonate derivative **1b** could be readily prepared and employed in the synthesis of **3a**; however, the bulk of the work described herein employs **3a** derived from **1a**.

Compound **3a**, thus prepared, is an odourless, bench-stable, free-flowing solid that forms an NHC film on Au(111) by simply immersing the Au surface in a methanol solution of **3a** in air at room temperature without any special precautions. These films have identical chemical and electrochemical stability to films deposited using the free carbene method^11^, resisting refluxing solvents, water, acid, base and oxidant (Supplementary Fig. 1)^11^.

### Film synthesis *in vacuo* and surface analysis

Benzimidazolium hydrogen carbonate **3a** was also used to generate films *in vacuo* from the solid state by connecting a solid doser containing **3a** to a ultra high vacuum chamber and warming to 325 K (Supplementary Fig. 2). Surface coverage is controlled by varying the amount of time the doser is open to the vacuum chamber. Monolayer formation is essentially complete after 5 min. Both the coverage and thermal stability of NHC were analysed using temperature programmed desorption (TPD) from the Au surface. Desorption profiles for fragments of various *m*/*z* are given in Supplementary Fig. 3, demonstrating that the NHC SAM has exceptional thermal stability. The *T*_max_ for loss of the NHC is 605 K, ∼125 K higher than simple thiols excluding interchain interactions (Fig. 1d)^20^. The absence of lower-temperature desorption peaks, especially at high surface coverage, indicates clean formation of a monolayer without physisorbed species.

From the *T*_max_, the NHC–Au bond strength is calculated to be 158±10 kJ mol^−1^ (ref. 21), consistent with our previously calculated bond strength of 149 kJ mol^−1^ (ref. 11). The lack of a substantial shift in the *T*_max_ with increasing surface coverage is consistent with weak inter-carbene interactions; therefore, this experiment directly probes the C–Au bond. Using a similar method, Bernasek and colleagues^20^ measured the chain length-independent bond energy for thiol films on gold to be 126 kJ mol^−1^.

NHC films were examined by high-resolution electron energy loss spectroscopy (HREELS) to probe NHC orientation and the C–Au bond. Figure 1e shows data from samples deposited at 300 K (blue) and annealed to 475 K (red), compared with the transmission mode infrared of the NHC gold chloride transition metal complex (orange) and a simulation of the components of the dipole moment derivative normal to the surface based on the calculated normal modes of NHC-Au-Cl (black). Notably, aromatic C–H stretching modes centred at 3,070 cm^−1^ show much greater relative intensity for the NHC on gold than in solution, consistent with **3a** binding perpendicular to the support, as only vibrational transitions with components of the dynamic dipole moment normal to the surface are observed in the dipolar scattering regime. After annealing to higher temperatures, the 750 cm^−1^ signal increases in intensity and all bands become more defined, consistent with molecules orienting in a more uniform and slightly tilted configuration after annealing. The Au–C stretch is observed at 420 cm^−1^. It is noteworthy that a similar stretch is not seen in the reference compound, as this is an Au(I) chloride compound and the Au–C bond thus appears at a different location.

**Figure 2:**
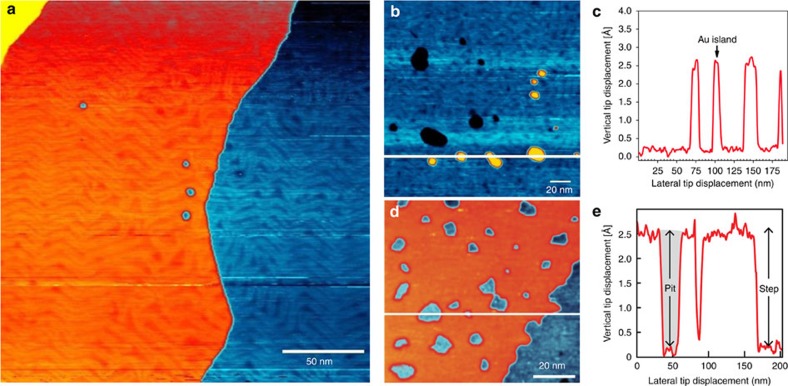
Scanning tunnelling microscopy imaging of NHC-based films. (**a**) Film prepared *in vacuo* from **3a** illustrating low pit density and adsorbate-modified herringbone reconstruction. (**b**) Lower magnification image of *in vacuo* deposited film illustrating pits and islands. (**c**) Line profile from **b**, showing 2.3 Å step height as expected for NHC-covered layers separated by a single height step of one gold atom. (**d**) Lower magnification image of solution-deposited film of **3a** illustrating pit density. (**e**) Line profile through pits from **d**.

Film uniformity is of critical importance for biosensing applications. This was probed by scanning tunnelling microscopy (STM). Films prepared *in vacuo* have high uniformity with subtle modification of the zigzag arrangement of soliton boundaries of the Au(111) herringbone reconstruction^22^ and very little islanding (Fig. 2a–c). Islands are one gold atom higher than terraced regions and pits are one atom lower, consistent with thiol- and phosphine-based SAMs^23^,^24^. SAMs prepared in methanol (Fig. 2d) are still of high quality, but with larger amounts of pitting and islanding compared with vapour-deposited films (Supplementary Figs 4–7). Largely intact herringbone structures can also be observed in solution-deposited films (Supplementary Fig. 4).

### Film analysis by electrochemical methods

**Figure 3:**
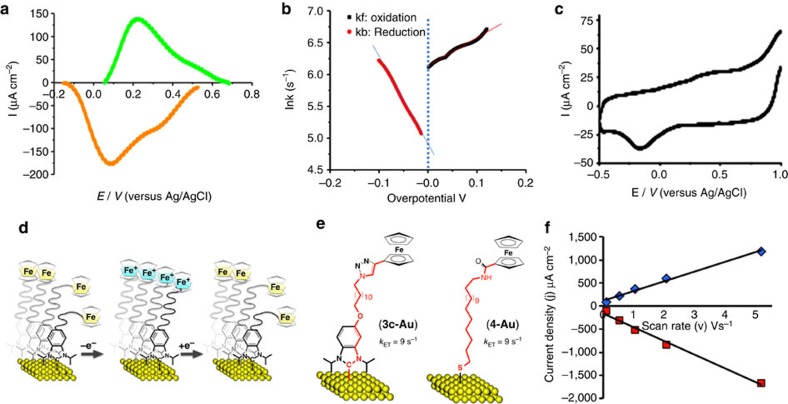
Electrochemical studies of NHC-based films. (**a**) Cyclic voltammetry (background subtracted) of **3c**-based films showing asymmetric electron transfer (scan rate 1 Vs^−1^). (**b**) Tafel plot. (**c**) Cyclic voltammogram measured at 200 Vs^−1^. (**d**) Restructuring of the film on oxidation to give a more ordered film with faster electron transfer. (**e**) Electron transfer rates of **3c**-based films compared with films from thiols of similar chain length. (**f**) Linear dependence of current density versus scan rate, confirming that the ferrocene conjugate is immobilized on the surface.

Surface coverage (film density) was probed quantitatively using electrochemical techniques by relating the Fc redox signal from **3c** to molecular density (Supplementary Fig. 9). Surface molecular density was determined to be 3.92±0.12 molecules per nm^2^. The cyclic voltammogram is asymmetric with regard to reduction and oxidation, with the reduction peak larger (Fig. 3a–c), an effect enhanced at faster scan rates (Fig. 3c). This may be interpreted as oxidized ferrocenyl groups forming a more ordered configuration (Fig. 3d), where they accept electrons more quickly through the film^25^. Tafel plots show the same effect (Fig. 3b)^26^. The average electron transfer rate, *K*_ET_, of 9.1 s^−1^ is remarkably similar to films prepared from FcCONH(CH_2_)_17_SH, with virtually the same number of atoms separating the gold and the ferrocene unit (9 s^−1^; Fig. 3e)^27^. The current density is shown to be linear with scan rate, indicating that the ferrocene moieties are immobilized (Fig. 3f).

### NHC films in SPR-based biosensing

**Figure 4:**
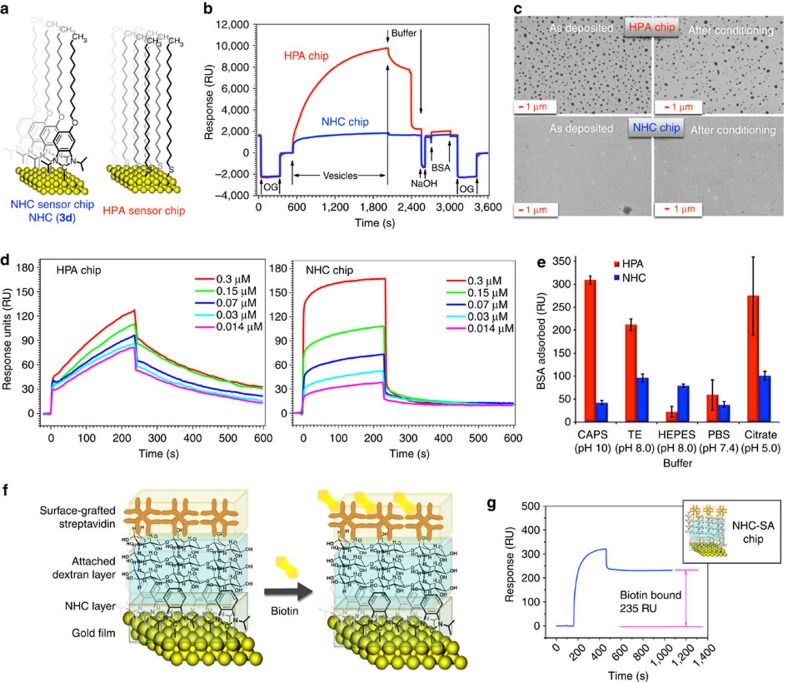
SPR-based biosensing with NHC chips. (**a**) Structures of NHC and HPA coatings. (**b**) Planar supported hybrid bilayer formation on NHC and HPA chips as monitored by SPR. (**c**) Analysis of chips from **b** by scanning electron microscopy, indicating vesicle presence on HPA chips. (**d**) Quantitative melittin sensing on the HPA (left) and NHC (right) chips. (**e**) Run-to-run variability in BSA adsorption on both chips as a function of buffer and pH. Average of four to eight runs in each case with standard deviation shown as a black bar. (**f**) Schematic illustration of biotin sensing on NHC-based dextran-linked version of streptavidin (NHC-SA) chip surface. (**g**) Response of biotin as observed on the NHC-SA chip surface.

Having confirmed that high-quality NHC films can be prepared easily in solution or *in vacuo*, we examined these films in SPR-based biosensing (Supplementary Figs 8 and 10)^28^,^29^. To enable as straightforward a comparison as possible, alkylated hydrogen carbonate **3d** was deposited on a clean gold SPR chip and benchmarked against the equivalent thiol-derived hydrophobic association chip (HPA) (Fig. 4a). The commercial HPA chip was chosen for initial studies, as it is composed of a simple alkylated thiol monolayer. This chip is typically employed to create supported hybrid bilayers^30^, which can then be used to interrogate receptor–analyte interactions in a membrane-like environment. When commercial HPA chips were treated with phosphatidylcholine vesicles (Fig. 4b), a large signal with substantial curvature is observed, attributed to the adsorption of a large excess of lipid, which is a typical behaviour for this chip^31^. Excess lipid is removed with a buffer rinse and base conditioning. In NHC-based films (Fig. 4b), complete formation of a supported hybrid bilayer is observed with almost no extraneous lipid adsorbed. Scanning electron microscopic analysis confirms this interpretation, showing what appear to be a significant number of vesicles on the thiol-based chip, even after conditioning (Fig. 4c top), with no evidence of vesicles on NHC-based films, even before conditioning. This may result from the greater spacing of NHC compared with the thiol, permitting efficient interdigitation of the phosphatidylcholine, whereas densely packed thiol SAMs probably require deformation before interdigitation can occur.

Adsorption of the lytic peptide melittin was then examined (Fig. 4d). This protein has been used in several cases, to benchmark hybrid-supported lipid bilayers^32^,^33^,^34^,^35^,^36^. For the HPA chip (left), the correlation between melittin concentration and response is poor and proper equilibration could not be achieved. By contrast, the NHC-based chip (right) shows a proportionate and reliable response to melittin concentration, permitting facile and accurate measurement of affinity constants^37^.

To determine conditions under which NHC-supported bilayers can operate, the quality of the supported bilayers was tested in the presence of different buffers at different pHs. Bovine serum albumin (BSA) absorption was employed as a benchmark for film quality, as it adsorbs at defects in the supported hybrid lipid bilayer^31^. Between each run, the chip was regenerated and a new lipid layer adsorbed to quantify run-to-run variability. As shown in Fig. 4e, in all cases except one, significantly greater amounts of BSA are adsorbed using the HPA chip compared with the NHC chip. As SPR-based biosensing is most powerful when performed using unprocessed biological samples, preventing the nonspecific adsorption of simple proteins is critical to prevent false positives. Diazonium-based films have provided advances in this area^38^, but suffer from difficulties controlling mono versus multilayer deposition^39^, which itself is a source of irreproducibility. The films reported herein were tested for BSA adsorption in a variety of buffers at different pHs (Fig. 4e) and show reproducible levels of nonspecific adsorption across the board with low standard deviation (s.d.) over four to eight runs. Compared with this, commercial thiol-based films show wide variance depending on the buffer employed. Most importantly, at pH extremes, the NHC chip dramatically outperforms the thiol-based HPA chip in terms of overall amount of BSA adsorbed and run-to-run variability. Furthermore, as melittin sensing (Fig. 4d) was carried out at neutral pH, it appears that during practical protein sensing, the NHC chip is superior even at neutral pH. Taken together, these results show improved behaviour at all pHs and open up the possibility for sensing in more extreme environments, which cannot be done using existing technology.

NHC chips also showed high thermal stability, surviving heating at 65 °C in air for 24 h (Supplementary Table 4). In addition, the NHC chip still gave reliable data within 9 months of use, whereas the commercial chip is reported to have a shelf life of only 3 months under nitrogen atmosphere.

With these preliminary proof-of-concept results in hand, we then prepared an NHC-linked version of a commonly employed carboxymethylated dextran and streptavidin (SA) chips, (Fig. 4f). To prepare these chips, the NHC is chemically derivatized with a pendant alcohol, which, through several chemical steps, is modified to permit attachment of a dextran layer. After carboxymethylation of this layer, the carboxymethylated dextran chip is characterized and the SA chip is then prepared by attaching SA using standard amine coupling procedures (see Supplementary Information section on Streptavidin). As shown in Fig. 4g, biotin could be easily detected using this chip in levels wholly consistent with commercial SA chips.

In conclusion, NHC films can be prepared in solution under ambient conditions and *in vacuo* from bench-stable, easily handled benzimidazolium hydrogen carbonates. Scanning tunnelling microscopy reveals that vapour-deposited NHC SAMs have exceptionally high quality with low pit and island density, and closely related morphological behaviour is observed following solution deposition. TPD and HREELS reveal that the NHC stands upright on the Au(111) surface and forms a very strong NHC-Au bond (∼158 kJ mol^−1^). Most importantly, NHC-based films provide significant advantages versus thiol analogs in SPR-based biosensing, including more reliable and reproducible bilayer formation, and more consistent protein sensing results under extremes of pH.

## Methods

### Synthesis of **3a** via hydrogen peroxide oxidation in presence of carbon dioxide

A 50 ml round-bottom flask capped with a rubber septum and containing a needle for ventilation and a glass pipette for addition of gaseous carbon dioxide was charged with a clear, colourless solution of 1,3-diisoproplylbenzimidazolium iodide (**1a**) (990.6 mg, 3 mmol) (ref. 11) in deionized water (30 ml) (pH 6). CO_2_ was bubbled through this solution for 1 min, after which time hydrogen peroxide (225 μl (30% w/v), 2.25 mmol in 0.5 ml water) was injected. Vigorous CO_2_ bubbling was maintained for 1 h under stirring during which time the solution turned yellow and then brown until the formation of a purple precipitate was detected. The mixture was filtered by vacuum filtration and washed with 3 ml of water resulting in a clear, colourless filtrate solution (pH 8), leaving the insoluble iodine as a violet solid precipitate. Water was removed by flushing air overnight over the surface of the solution then the product was dried under high vacuum for 2 h to give a white solid. The resulting solid was triturated and sonicated in acetone (3 × 3 ml), which was then decanted off. Subsequent drying under vacuum afforded the desired product as a white powder (478 mg, 66% yield). It is worthwhile to note that this procedure cannot be applied in organic solvents such as methanol due to the solubility of the formed iodine and its disproportionation under basic conditions.

To test for complete removal of iodine, a qualitative silver nitrate test was performed where one drop from the reaction aliquot was mixed with excess aqueous silver nitrate (1 M) solution. In cases where incomplete exchange was observed, a yellow precipitate of silver iodide formed that persisted on the addition of nitric acid. When iodide was completely exchanged, a white precipitate of silver bicarbonate formed that became colourless on addition of a solution of 1 M nitric acid. Quantitatively, the removal of iodide was assessed by elemental analysis in house looking at CHN and also externally analysing for iodide content. Mp: 123–124 °C (dec.).

### Synthesis of **3a** via hydrogen carbonate-anion exchange resin

A hydrogen carbonate exchange resin was prepared from commercial (Sigma Aldrich) Amberlyst A26 hydroxide resin. To accomplish the conversion, 10 g (0.8 meq ml^−1^) of the resin was suspended in 10 ml deionized water (pH 8) and carbon dioxide bubbled through the solution for 0.5 h (pH 6, as measured by a pH strip). To test the conversion of the resin, aqueous KI (0.2 ml, 0.4 M) was added to 0.2 ml of resin before and after CO_2_ bubbling. The mixtures were sonicated for 10 min and to one drop of the aliquots, excess aqueous silver nitrate (1 M) was added. The fresh (hydroxide) resin gave a dark brown precipitate of silver oxide, whereas the bicarbonate resin gave a white precipitate of silver bicarbonate. Both precipitates gave a clear colourless solution after addition of nitric acid.

Resin-HCO_3_ suspended in water was measured out in a graduated cylinder (3.8 ml, 3 equiv., prepared as described above) and transferred to a 20 ml vial where the resin was allowed to settle and water was decanted. The resin was washed with methanol (3 × 2 ml). 1,3-Diisoproplylbenzimidazolium iodide (**1a**) (330 mg, 1 mmol) was dissolved in 5 ml methanol and transferred to the resin. The mixture was stirred for 30 min. The silver nitrate test indicated the completeness of the exchange reaction. The hydrogen carbonate solution was passed through a cotton plug to remove any resin beads and the resin was washed with methanol (3 × 2 ml), which was then added to the original filtrate. Solvent was evaporated and the residual solid was triturated and sonicated in acetone (3 × 3 ml), which was then decanted off via syringe and discarded. Subsequent drying of the white powder under vacuum afforded the desired product as a white powder (198 mg, 75% yield). Mp: 123–125 °C (dec.).

### Preparation of SAMs

Before functionalization, the Au(111) films were cleaned by washing the films in 3 × 2 ml of methanol, drying them under an argon gas (4.8 Praxair) stream for 1 min, then cleaning them with plasma generated from room air at a medium radio frequency (RF) level and a pressure kept between 300 and 500 mtorr for 1 min. The films were then used immediately for functionalization.

### Film formation from free carbenes in solution

SAMs were prepared by immersion of Au(111) on mica substrates in a 1 mM solution of the corresponding free carbene^14^ dissolved in dry toluene at room temperature in the glove box. Substrates were then rinsed in toluene (10 × 2 ml) and dried under an argon gas (4.8 Praxair) stream for 1 min.

### Film formation from hydrogen carbonates in solution

SAMs were prepared by immersion of Au(111) on mica substrates in 1 to 10 mM solutions of the corresponding benzimidazolium hydrogen carbonate salt in methanol for 24 h at room temperature in air. Substrates then were rinsed in methanol (5 × 2 ml) and dried under an argon gas (4.8 Praxair) stream for 1 min.

### Film formation via vapour deposition

*i*Pr_2_bimy(H)[HCO_3_] (**3a**) was outgassed for 12 h at room temperature on a differentially pumped manifold separated from the ultra high vacuum system by a gate valve. Dosing was achieved by resistively heating a glass microcapillary wrapped in Ta wire containing compound **3a** (Supplementary Fig. 2). Calibration of dosing temperature was achieved by gradually increasing the temperature until the mass spectrum displayed a fragmentation pattern consistent with the presence of the molecule in the gas phase. TPD experiments were conducted for various exposure times at a heating rate of *β*=3.5 K s^−1^. Coverages are reported as fractions of a full monolayer. One monolayer (1 ML) is defined as the total integrated area of the peak for the fragment *m*/*z*=41 amu (most intense) achieved on saturation exposure (adsorption beyond a chemisorbed monolayer was not observed at 300 K). Subsequent coverages are expressed as the area under the curve relative to the saturated peak area. The best fit accompanying the data was achieved by the sum of Gaussian functions and a 10–15 point baseline. HREELS spectra were collected in specular mode (*θ*_i_=*θ*_f_=45°) at 20 scans per spectrum, with a primary beam energy of *E*_0_=5 eV and at a resolution of 3.85 meV measured as the full width at half maximum of the elastic peak. All spectra have been normalized to the elastic peak.

*i*Pr_2_bimy(H)[HCO_3_] (**3a**) was dosed on the Au (111) crystal, which was held at room temperature. Multiplexing between 1 and 150 amu in TPD experiments revealed fragments *m*/*z*=2, 27, 39 and 41 amu desorbing from the surface. No other signal was detected that could indicate co-adsorption of the carbonate counterion (*m*/*z*=60, 44 or 28 amu) nor any other higher mass fragment.

### Data availability

Spectral and purity data are available for all new compounds, along with original X-ray photoemission spectroscopy data, SPR data, TPD, HREELS, low-energy electron diffraction and electrochemical data. All data described in the manuscript are available from the authors upon request.

## Additional information

**How to cite this article:** Crudden, C. M. *et al.* Simple direct formation of self-assembled N-heterocyclic carbene monolayers on gold and their application in biosensing. *Nat. Commun.* 7:12654 doi: 10.1038/ncomms12654 (2016).

## Supplementary Material

Supplementary Information Supplementary Figures 1-10, Supplementary Tables 1-5, Supplementary Methods and Supplementary References
