# Supplementary material for: Simple direct formation of self-assembled N-heterocyclic carbene monolayers on gold and their application in biosensing
Source: Nat Commun. 2016 Sep 2;7:12654. doi: 10.1038/ncomms12654 (PMC5025784; doi:10.1038/ncomms12654)
Supplement: Supplementary Information — Supplementary Figures 1-10, Supplementary Tables 1-5, Supplementary Methods and Supplementary References [file ncomms12654-s1.pdf]

Supplementary Figures

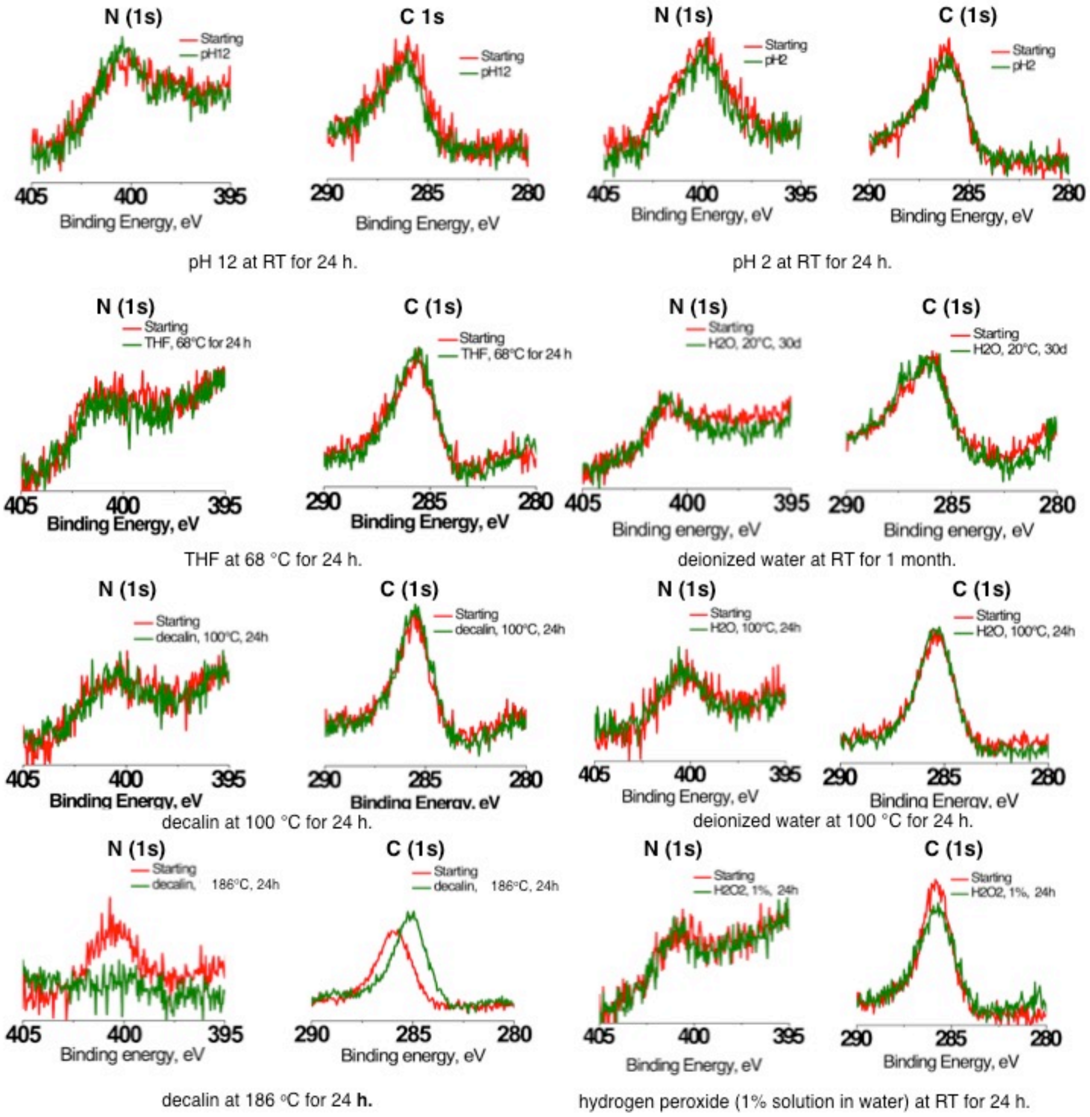

Supplementary Figure 1: NHC films deposited on Au(111) from *i*Pr_2bimy(H)[HCO_3] (3a) before (red) and after (green) exposure to a variety of conditions.

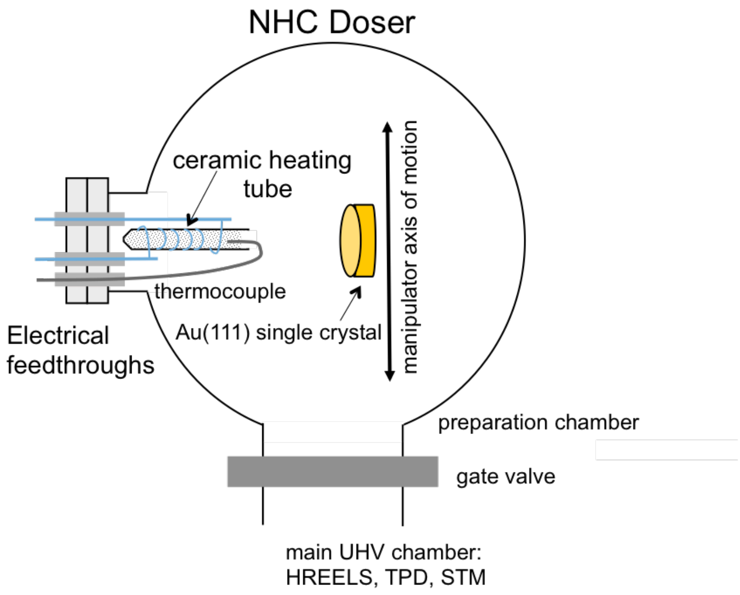

Supplementary Figure 2: Dosing set up

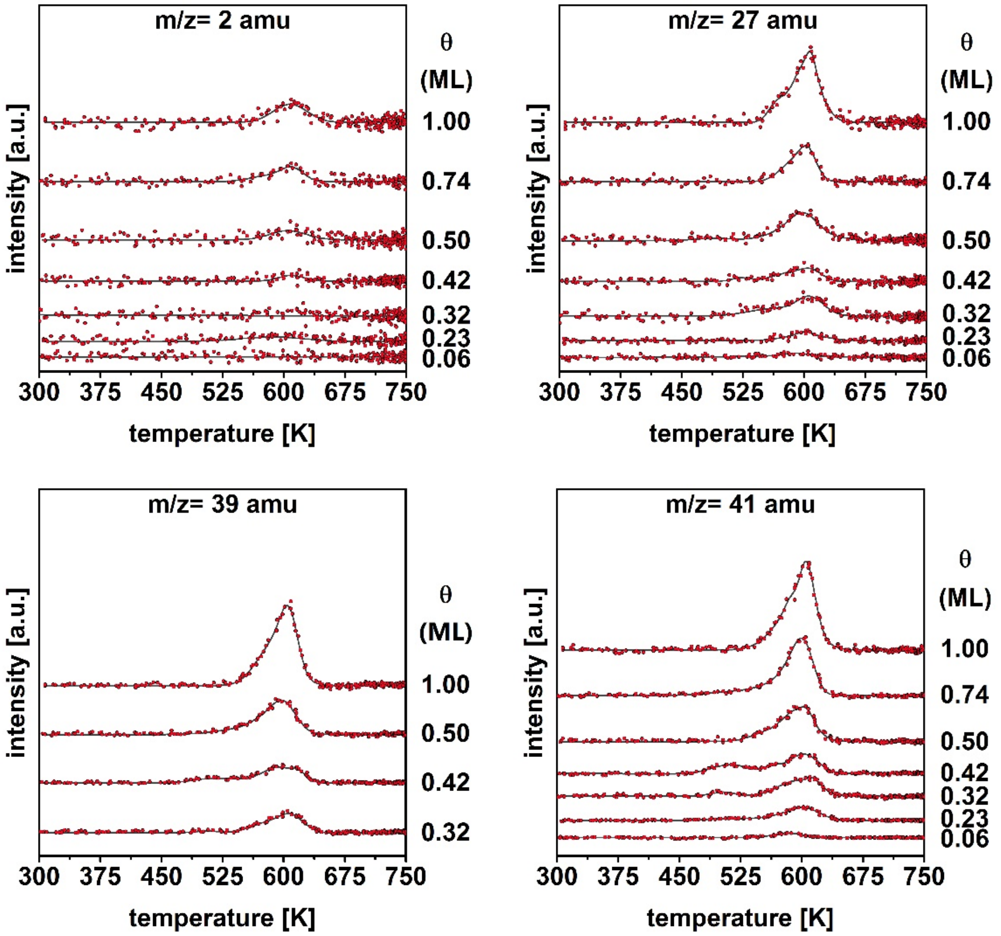

Supplementary Figure 3: TPDs showing the traces for the fragment m/z = 2, 27, 39, and 41 amu of *i*Pr_2bimy adsorbed at room temperature on Au(111). The TPD traces have been offset manually, and the intensity is arbitrary.

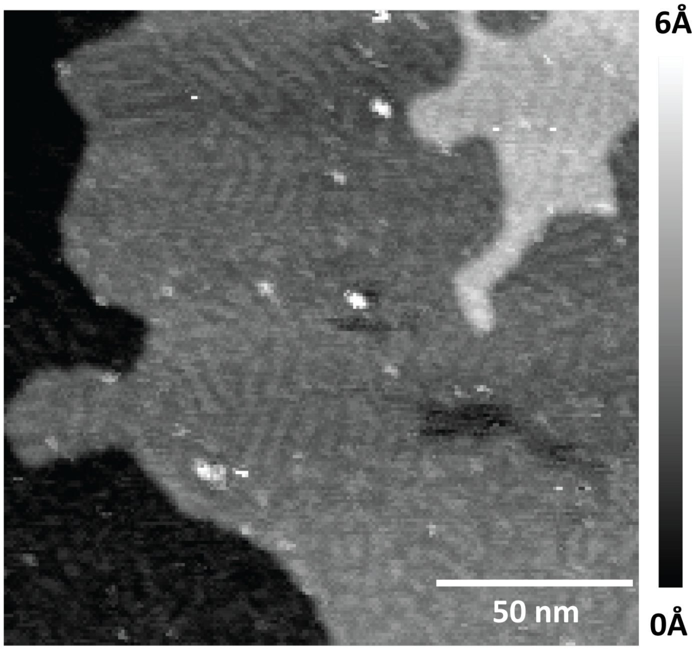

Supplementary Figure 4: Altered herringbone reconstruction on Au(111) due to adsorption of NHC **2b** on the surface from solution grafting of **3b**. Image details: 160 nm x 160 nm, bias voltage = 0.5 V and set current 5 pA.

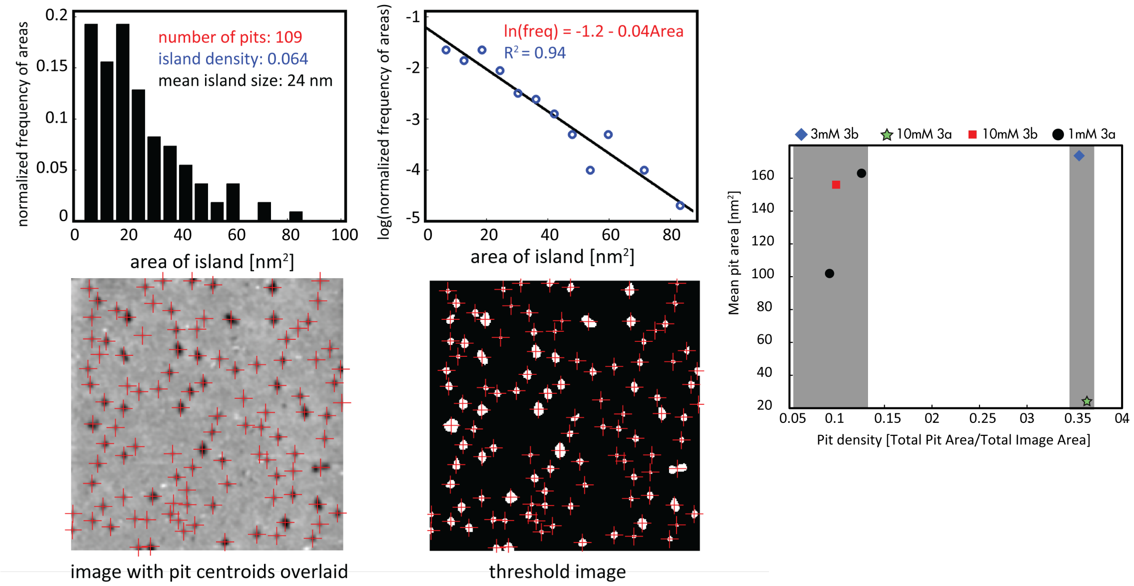

Supplementary Figure 5: Result of threshold analysis for a typical molecule on Au(111) sample and mean pit size across different molecules. Pit density appears to occur in two regimes.

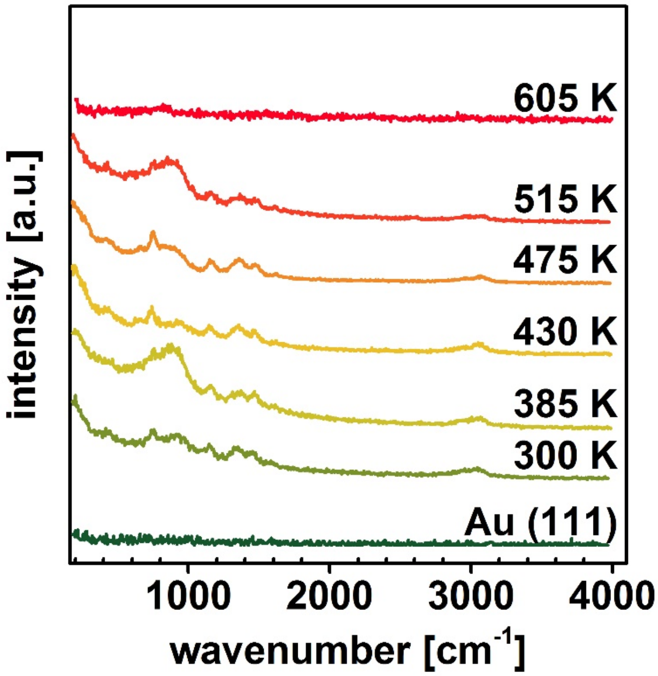

Supplementary Figure 6: HREEL spectra of iPr_2bimy at saturation exposure on Au (111) at 300K and annealed to the designated temperatures.

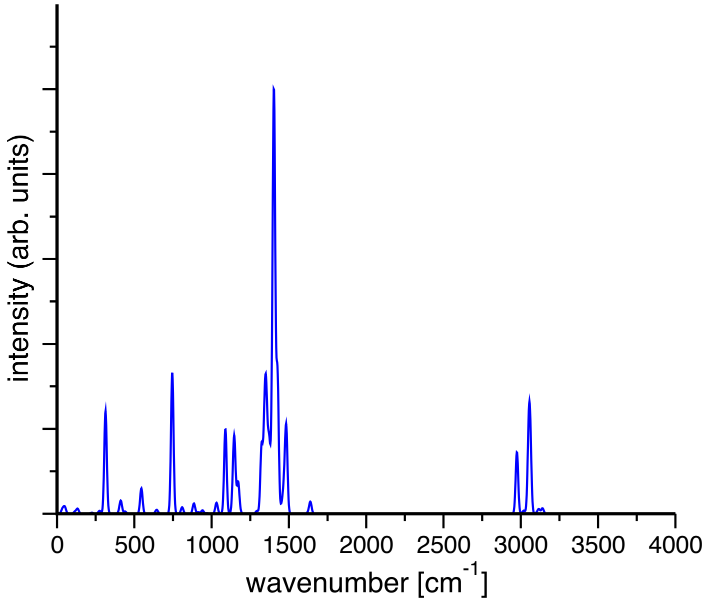

Supplementary Figure 7: Simulated IR spectrum of an NHC-Au-Cl complex of iPr_2bimy using M06-2X functional in conjunction with the SDD effective core potential/basis set for Au and a TZVP basis set for all other atoms.

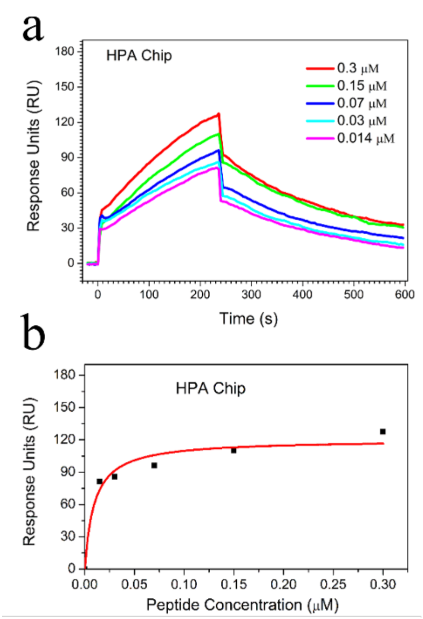

Supplementary Figure 8. Sensorgrams of binding between various concentrations of melittin (0.015 μM to 0.3μM) and the lipid monolayers on HPA and NHC sensor chips (a). The corresponding relationship between the equilibrium binding responses (RU_{eq}) and melittin concentrations (b). The data were fitted using the steady-state affinity model (red lines).

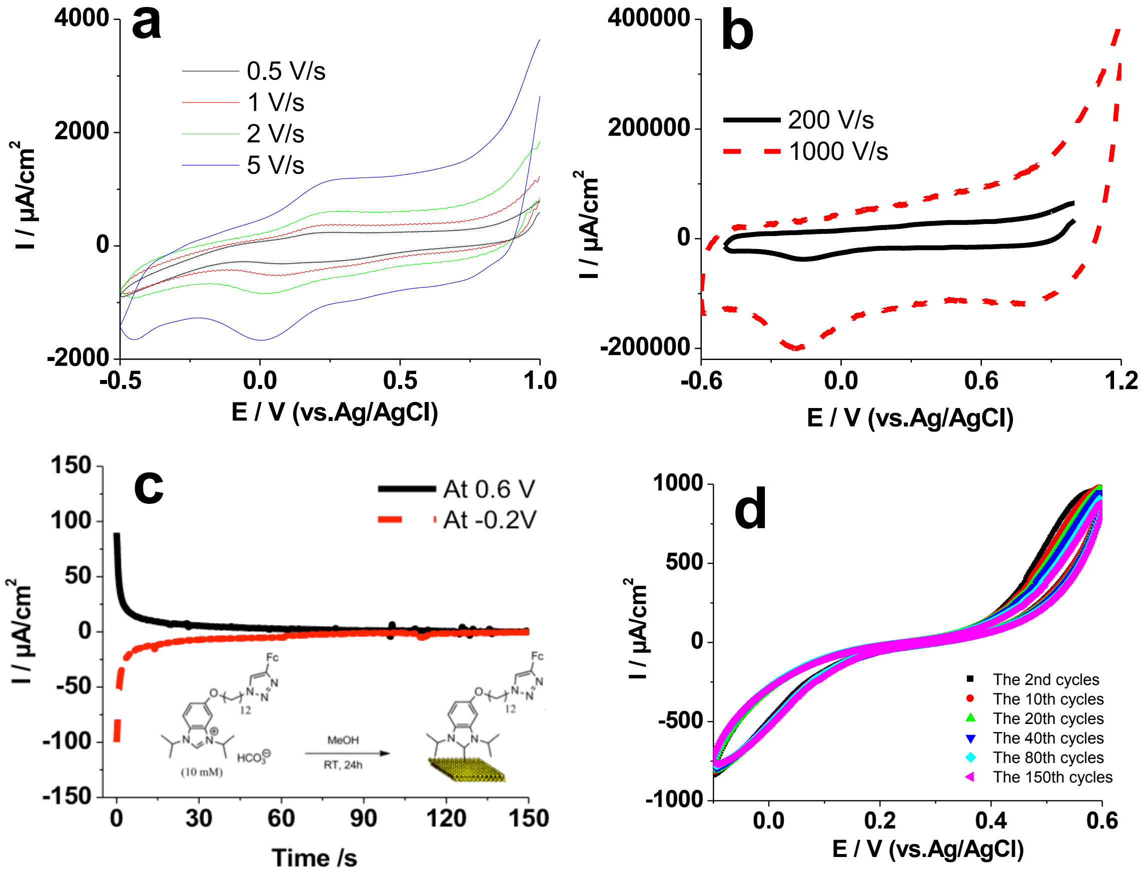

Supplementary Figure 9: (a) & (b) Cyclic voltammograms of immobilized Fc group via dipropylcarbene on gold surface at different scan rates. The measurements were carried out in 0.1 M $NaClO_4$ aqueous solutions. (c) Current vs time measurement of immobilized Fc group via 3c on gold surface. The measurements were carried out in 0.1 M $NaClO_4$ aqueous solutions. (d) Electrochemical cycling of 3d over 150 cycles in 5 mM/5 mM $Fe(CN)_6^{3-/4-}$ aqueous solution with 1 M $NaClO_4$ as supporting electrolyte.

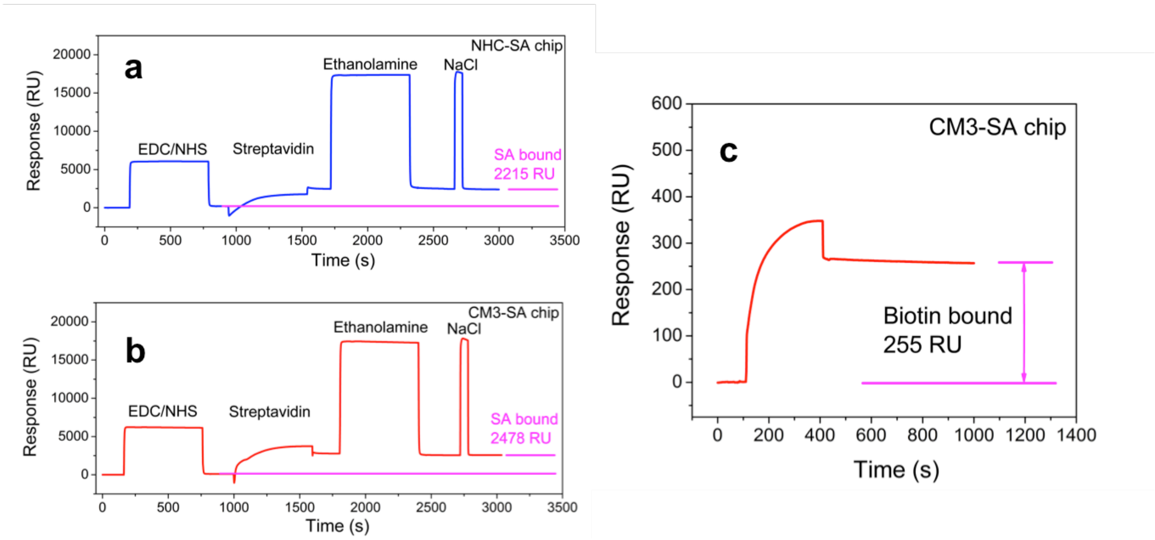

Supplementary Figure 10. Sensorgrams for the immobilization of streptavidin (0.1 mg/ml, in 10 mM Sodium Acetate, PH 4.7) using a standard amine-coupling method on NHC-CM (a) and Biacore CM3 (b) surfaces. The sensorgram for CM3-SA chip to bind D-biotin (c).

Supplementary Tables

Supplementary Table 1: XPS Analysis of select hydrogen carbonate salts deposited on Au(111)

Molecule	Predicted C:N(:Ir)	Observed C:N(:Ir)
*i*Pr$_2$bimy(H)[HCO$_3$] (**3a**)	13:2	13:2
5-((12-(4-(ferrocenyl)-1H-1,2,3-triazol-1-yl)dodecyl)oxy)-1,3-diisopropyl-1H-benzo[d]imidazol-3-ium hydrogen carbonate (**3c**)	37:5:1	34:5:5
5-(dodecyloxy)-1,3-diisopropyl-1H-benzo[d]imidazol-3-ium hydrogen carbonate (**3d**)	25:2	27:2

Supplementary Table 2: HREELS spectra modes assignment. υ: stretch, ω: wagging, σ: scissoring, β: bending, ρ: rocking def.: deformation

**Energy loss (cm^{-1})**	**Assignment**
420	υ(C-Au)
650	ring stretch
750	aromatic ω(CH)
870	υ(C-$(CH_3)_2$)
930	υ(C-$(CH_3)_2$)
1015	ring breathing
1155	υ(C-$(CH_3)_2$)) + ρ(C-H)
1240	υ(CN)
1360	υ(CC) + υ(CN) + β(C-H)
1465	υ(CC) + υ(CN) + β(C-H) + ringdef.
1615	ring def. + σ(CH)
2960	υ(CH) isopropyl
3070	υ(CH) aromatic

Supplementary Table 3: Effect of pH conditions on hybrid lipid bilayer formation on both the commercial HPA SPR sensor chip and our NHC-derived SPR sensor chip.

**Buffer**[a]	**pH**	**Sensor chip**	**Initial loading (RU)**	**Loading after NaOH wash (RU)**	**BSA bound (RU)**
Citrate[b]	5.0	**HPA**	3924 ± 222 (6 %)	2598 ± 145 (6 %)	274 ± 85 (31 %)
		**NHC**	1893 ± 50 (3 %)	1449 ± 31 (2 %)	101 ± 9 (9 %)
PBS[c]	7.4	**HPA**	9577 ± 365 (4 %)	1596 ± 29 (2 %)	59 ± 33 (56 %)
		**NHC**	2330 ± 115 (5 %)	1724 ± 153 (9 %)	38 ± 6 (16 %)
HEPES[b]	8.0	**HPA**	2600 ± 543 (21 %)	1348 ± 24 (2 %)	22 ± 12 (55 %)
		**NHC**	1244 ± 26 (2 %)	909 ± 32 (4 %)	79 ± 4 (5%)
TE[b]	8.0	**HPA**	804 ± 33 (4 %)	516 ± 40 (8 %)	212 ± 13 (6 %)
		**NHC**	937 ± 30 (3 %)	592 ± 27 (5 %)	96 ± 4 (4%)
CAPS[b]	10.0	**HPA**	846 ± 32 (4 %)	451 ± 14 (3 %)	309 ± 9 (3 %)
		**NHC**	1366 ± 18 (1 %)	939 ± 33 (4 %)	42 ± 5 (12 %)

[a]Buffer abbreviations are as follows: **Citrate**: 100 mM citric acid monohydrate, 200 mM NaH_2PO_4; **PBS**: 100mM Na_2HPO_4/NaH_2PO_4, 150 mMNaCl; **HEPES**: 10 mM N-(2-hydroxyethyl) 1-piperazine-N'-(2-ethanesulphonic acid), 100 mM NaCl; **TE**: 10 mM tris(hydroxymethyl)aminomethane, 1 mM disodium ethylenediaminetetraacetate; **CAPS**: 10 mM 3-(cyclohexylamino)-1-propanesulfonic acid, 150 mM NaCl. [b]Values given as response units with standard deviations and relative standard deviations for n=4. [c]Values given as response units with standard deviations and relative standard deviations for n=8.

Supplementary Table 4: Effect of heating our NHC-derived SPR sensor chip at 65°C in air for 24 hours on hybrid lipid bilayer formation.[a]

**Buffer**	**pH**	**NHC chip**	**Initial loading (RU)**	**Loading after NaOH wash (RU)**	**BSA bound (RU)**
PBS	7.4	before heating	1671±41 (2.5 %)	1325±34 (2.6 %)	54±8 (15 %)
		after heating	1880 ± 22 (1.2 %)	1364 ± 54 (4.0 %)	61 ± 9 (15 %)

[a]Values given as response units with standard deviations and relative standard deviations for n=4.

Supplementary Table 5: Equilibrium Affinity Constants of the Peptides with PC/Cholesterol on sensor chips HPA, carbene, and L1. Derived According to a Steady-State Affinity Model.

PC/Cholesterol (10:1 w/w) K_A ($\times 10^6$ M^{-1})				
Sensor Chip	HPA chip[31] (monolayer)	HPA chip (monolayer) This work	NHC chip (monolayer) This work	L1 chip[31] (bilayer)
melittin	0.0188	105	7	0.47

Supplementary Methods

Abbreviations

iPr_2bimy	(**2a**, 1,3-dihydro-1,3-bisisopropylbenzimidazol-2-ylidene)
iPr_2bimy(H)[HCO_3]	(**3a**, 1,3-diisopropylbenzimidazolium hydrogen carbonate)
UHV	ultra high vacuum
TPD	temperature programmed desorption
LEED	low energy electron diffraction
HREELS	high resolution electron energy loss spectroscopy
STM	scanning tunneling microscopy
XPS	X-ray photoelectron spectroscopy
SAM	self assembled monolayer
SPR	surface plasmon resonance
THF	tetrahydrofuran
NMR	nuclear magnetic resonance
gs-COSY	gradient selected correlation spectroscopy
gs-HSQC	gradient selected heteronuclear single-quantum correlation
gs-HMBC	gradient selected heteronuclear multiple bond correlation

Materials and Methods

General Considerations

Solvents were used without purification except where stated. THF was distilled from sodium, and DriSolv® methanol was purchased from EMT and used as received. All other reagents were purchased from chemical suppliers and used as received. Reactions requiring an inert atmosphere were carried out in a nitrogen-filled glovebox (M. Braun) with oxygen and water levels ≤2 ppm.
STM measurements were also performed in UHV at room temperature using a lab-built Pan-style STM [1] on molecules added from solution to commercial Au(111) on mica surfaces. Mechanically formed platinum-iridium tips were used for all experiments. GXSM [2] was used as control software using the Signal Ranger A810 DSP and Nanonis HVA4 high-voltage amplifier.

All electrochemical measurements were performed with three-electrode configuration electrochemical cell, which was in an enclosed Faraday cage, and a CHI-660b potentiostat (CH Instruments, Austin, TX). A reference electrode (Ag/AgCl/3.0 M KCl), a platinum counter electrode and a salt bridge were used for all the experiments. The salt bridge was filled with agar solution, prepared by dissolving 2 grams of agar and 10.1 g KNO_3 in 100 mL of water. The solutions are always purged thoroughly with argon for 20-30 minutes and all the experiments were carried out in an Argon protected environment. Two types of electrolytes have been used. An aqueous solution of 0.1 M $NaClO_4$ and an aqueous solution of 5 mM/5 mM $Fe(CN)_6^{3-/4-}$ with 1 M $NaClO_4$ as supporting electrolyte were used as the electrolytes for electrochemistry on Fc-labeled films and electrochemical cycling tests respectively.

Instrumentation

^{1}H and ^{13}C NMR, spectra were recorded on Bruker Avance-400, 500 or 600 MHz spectrometers. Chemical shifts are reported in delta (δ) units, expressed in parts per million (ppm) downfield from tetramethylsilane, using residual protonated solvent as an internal standard (^{1}H NMR $CDCl_3$: 7.26, methanol-*d*$_4$: 3.31 ppm; ^{13}C NMR $CDCl_3$: 77.16, methanol-*d*$_4$: 49.00 ppm).$CFCl_3$ (0.00 ppm) was used as an external standard for ^{19}F NMR. All 2D spectra (gs-COSY, gs-HSQC, gs-HMBC) were acquired in the phase-sensitive mode. All data were acquired, processed, and displayed using BrukerXWinNMR and MestReNova software and a standard pulse-sequence library. All measurements were carried out at 298 K.
IR spectra were collected on a Bruker ALPHA Platinum ATR as neat solids and absorption bands are given in cm^{-1}. Melting points were recorded on an Electrothermal MEL-TEMP apparatus connected to a Fluke 51 II Thermometer. Temperatures are given in degree Celsius (°C) and are uncorrected. Mass-spectrometry was carried out using a Micromass Platform LCZ 4000 system. Elemental analyses were performed using Flash 2000 CHNS-O analyzer or Carlo Erba EA 1108 CHNOS Elemental Analyzer.
A plasma cleaner (Harrick Plasma Cleaner/Sterilizer PDC-32G, Ossining, NY) was used. TGA experiments were performed using TGA Q500 with platinum crucibles. A constant heating rate of 5°C/min and gas purging (N_2) at a flow rate of 60 mL/min were used. The amount of samples used for TGA was between 10 and 11 mg.
XPS measurements were performed using a Thermo Microlab 310F ultrahigh vacuum (UHV) surface analysis instrument using Al Kα X-rays (1486.6 eV) or Mg Kα (1253.4 eV) at 15 kV anode potential and 20 mA emission current with a surface/detector take off angle of 75°. The binding energies of all spectra were

calibrated to the Au 4f line at 84.0 eV. A Shirley algorithm was used as the background subtraction method for all peaks. The Powell peak-fitting algorithm was used, with peak areas normalized between different elements using the relative XPS sensitivity factors of Scofield.[3] In cases where absolute peak intensities for a single element were compared between different samples, care was taken to ensure a standard sample size and orientation with respect to the X-ray source and detector within the analysis chamber. Calibration of our system using Au thiol SAMs of known surface concentration gave peak areas reproducible within ± 5% between sample runs.

Calculations

Frequency calculations of an NHC-Au-Cl complex related to **2a** were performed using Kohn-Sham density functional theory [4,5] with the M06-2X exchange-correlation functional [6] in conjunction with the TZVP basis set [7] for C, N, Cl, and H and the SDD basis set/effective core potential combination [8,9] for Au. The calculated frequencies were shifted using a scaling factor of 0.97.[10] The components of the dipole moment derivatives along the direction of the Au-C bond were required for each normal mode and used to generate the simulated infrared spectrum of **2a** residing perpendicular to an Au surface.

Synthetic Methods

1,3-Diisopropylbenzimidazolium iodide, **1a**, was prepared according to literature procedures.[11]

1,3-Diisopropylbenzimidazolium hydrogen carbonate, [*i*Pr_2bimy (H)][HCO_3] (3a)

Method 1: Hydrogen peroxide oxidation in presence of carbon dioxide

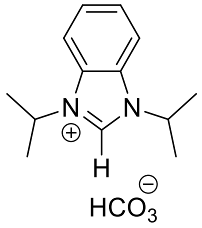

(3a)

A 50 mL round bottom flask capped with a rubber septum and containing a needle for ventilation and a glass pipette for addition of gaseous carbon dioxide was charged with a clear colorless solution of 1,3-diisoproplylbenzimidazolium iodide (**1a**) (990.6 mg, 3 mmol) (*10*) in deionized water (30 mL) (pH=6). CO_2 was bubbled through this solution for 1 min, after which time hydrogen peroxide (225 µL (30% w/v), 2.25 mmol in 0.5 mL water) was injected. Vigorous CO_2 bubbling was maintained for 1 h under stirring during which time the solution turned yellow and then brown until the formation of a purple precipitate was detected. The mixture was filtered by vacuum filtration and washed with 3 mL of water resulting in a clear colorless filtrate solution (pH = 8), leaving the insoluble iodine as a violet solid precipitate. Water was removed by flushing air overnight over the surface of the solution then the product was dried under high vacuum for 2 h to give a white solid. The resulting solid was triturated and sonicated in acetone (3 x 3 mL), which was then decanted off. Subsequent drying under vacuum afforded the desired product as a white powder (478 mg, 66% yield). It is worthy to note that this procedure can not be applied in organic solvents such as methanol due to the solubility of the formed iodine and its disproportionation under this basic condition.

To test for complete removal of iodine, a qualitative silver nitrate test was performed where one drop from the reaction aliquot was mixed with excess aqueous silver nitrate (1 M) solution. In cases where incomplete exchange was

observed, a yellow precipitate of silver iodide formed that persisted upon the addition of nitric acid. When iodide was completely exchanged, a white precipitate of silver bicarbonate formed that became colorless upon addition of a solution of 1M nitric acid. Quantitatively, the removal of iodide was assessed by elemental analysis in house looking at CHN and also externally analyzing for iodide content. Mp: 123-124 °C (dec.).

^{1}H NMR (400 MHz, CD_3OD): δ 8.07 (dd, J = 6.3, 3.2 Hz, 2H, Ar-H), 7.75 (dd, J = 6.3, 3.1 Hz, 2H, Ar-H), 5.11 (hept, J = 6.7 Hz, 2H, NC*Hi*Pr), 1.77 (d, J = 6.7 Hz, 12H, C*H₃i*Pr). The N_2CH and HCO_3 protons could not be observed due to their rapid exchange with the deuterated solvent on the NMR time scale.
^{13}C (^{1}H) NMR (101 MHz, CD_3OD): δ 161.42 (s, HCO_3^-), 138.96 (s, N_2*C*H), 132.59 (s, C_q), 128.19 (s, C_{Ar}), 114.97 (s, C_{Ar}), 52.83 (s, *C*H*i*Pr), 22.11 (s, *C*H_3*i*Pr).
ATR-IR: strong peaks for CO_2 asym. str. at 1622 cm^{-1} and sym. str. at 1367 cm^{-1}
Anal. Calc. for $C_{14}H_{20}N_2O_3$: C, 63.62; H, 7.63; N, 10.60. Found: C, 63.63; H, 7.83; N, 10.38, I < 0.2.
HRESI-MS (m/z) for $C_{13}H_{19}N_2^+$ $[M-HCO_3]^+$: 203.1543, Calc.: 203.1543.

Method 2: Hydrogen carbonate-anion exchange resin

Firstly, a hydrogen carbonate exchange resin was prepared from commercial (Sigma Aldrich) Amberlyst A26 hydroxide resin. To accomplish the conversion, 10 g (0.8 meq/mL) of the resin was suspended in 10 mL deionized water (pH = 8) and carbon dioxide bubbled through the solution for 0.5 h (pH = 6, as measured by a pH strip). To test the conversion of the resin, aqueous KI (0.2 mL, 0.4 M) was added to 0.2 mL of resin before and after CO_2 bubbling. The mixtures were sonicated for 10 min and to one drop of the aliquots, excess aqueous silver nitrate (1 M) was added. The fresh (hydroxide) resin gave a dark brown precipitate of silver oxide, while the bicarbonate resin gave a white precipitate of silver bicarbonate. Both precipitates gave a clear colourless solution after addition of nitric acid. After this, the resin was used to treat several iodide salts as described below.

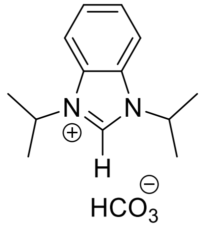

(3a)

Resin-HCO_3 suspended in water was measured out in a graduated cylinder (3.8 mL, 3 equiv., prepared as described above) and transferred to a 20 mL vial where the resin was allowed to settle and water was decanted. The resin was washed with methanol (3 x 2 mL). 1,3-Diisoproplylbenzimidazolium iodide (**1a**) (330 mg, 1 mmol) (*10*) was dissolved in 5 mL methanol and transferred to the resin. The mixture was stirred for 30 min. The silver nitrate test indicated the completeness of the exchange reaction. The hydrogen carbonate solution was passed through a cotton plug to remove any resin beads and the resin was washed with methanol (3 x 2 mL), which was then added to the original filtrate. Solvent was evaporated and the residual solid was triturated and sonicated in acetone (3 x 3 mL), which was then decanted off *via* syringe and discarded. Subsequent drying of the white powder under vacuum afforded the desired product as a white powder (198 mg, 75% yield). Mp: 123-125 °C (dec.).

Spectra were consistent with compound prepared using the method described in 3.1.1.

Anal. Calc. for $C_{14}H_{20}N_2O_3$: C, 63.62; H, 7.63; N, 10.60. Found: C, 63.34; H, 7.70; N, 10.71

Method 3: Hydrogen carbonate-anion exchange resin with triflate starting material

.

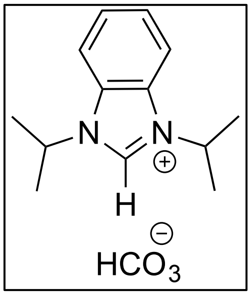

Amberlyst A26 resin in water was bubbled with CO_2 gas for 15 minutes to regenerate the resin with HCO_3^- anions. An aliquot of resin-HCO_3 (3.2 mL, 2.6 mmol) was taken to a 20 mL-vial and water was pipetted off. Resin-HCO_3 was washed three times with MeOH (2 mL) and solution of 1,3-diisopropylbenzimidazolium triflate (**1b**, 0.3 g, 0.85 mmol) in MeOH (5 mL) was added and the mixture was stirred at RT at medium speed for 30 minutes. Solution was collected and filtered through a cotton plug, and solvent was removed by rotavap. The resulting oily residue was dried in vacuum affording white solid. The crude product was washed successively with acetone and dried in vacuum (0.2 g, 75 %).

Spectra matched those shown above. Anal. Calc. for $C_{14}H_{20}N_2O_3$: C, 63.62; H, 7.63; N, 10.60. Found: C, 63.74; H, 7.74; N, 10.55.

1-Isopropylbenzimidazole.

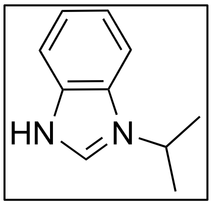

A mixture of benzimidazole (1.0 g, 8.7 mmol), anhydrous Cs_2CO_3 (4.4 g, 13.6 mmol) and 2-bromopropane (2.2 mL, 23.4 mmol) in acetonitrile (60 mL) was refluxed overnight with stirring. After cooling to RT, solvent was removed. Dichloromethane was added to the residue and the resulting mixture was filtered by suction through a pad of Celite and washed a few times with dichloromethane. Solvent was removed *in vacuo* yielding a yellowish liquid, which was further purified by silica column chromatography with dichloromethane-MeOH 95:5 mixture (R_f = 0.59 in dichloromethane -MeOH 9:1). The product was obtained as pale yellow liquid (1.3 g, 92%).

Spectra were consistent with the literature.[12]

(3.3) 1,3-Diisopropylbenzimidazolium triflate (1b).[13]

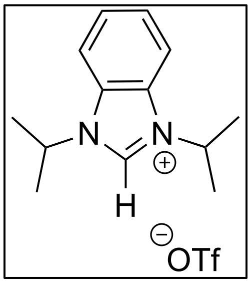

To a stirred solution of anhydrous 2-propanol (0.43 mL, 5.6 mmol) and anhydrous pyridine (0.45 mL, 5.6 mmol) in dry dichloromethane (10 mL) at −20°C under argon was slowly added triflic anhydride (0.92 mL, 5.6 mmol), which resulted in a formation of white precipitate. The reaction mixture was stirred at −20°C for 30 minutes and then at RT for additional 30 minutes. Pentane (10 mL) was added to further precipitate the formed salts and reaction mixture was then filtered by suction through a pad of Celite to a solution of 1-isopropylbenzimidazole (0.6 g, 3.8 mmol) in dry dichloromethane (10 mL). Reaction mixture was stirred under argon at −10°C for 1 hour and then at RT overnight. Dichloromethane was removed *in vacuo* to yield slightly pink viscous oil. The product was purified either by silica column chromatography (dichloromethane-MeOH, 95:5) or by trituration depending on the impurities. The crude product was dissolved in small amount of dichloromethane and cooled to −10°C. Diethyl ether was slowly added to the

stirred solution to precipitate product as white solid. The product was collected and washed with diethyl ether and dried in air (0.9 g, 69 %).

^{1}H NMR (400 MHz, $CDCl_3$): δ 9.84 (s, 1H, N-C*H*=N), 7.81 (dd, J = 6.4, 3.2 Hz, 2H, Ar-H), 7.66 (dd, J = 6.4, 3.1 Hz, 2H, Ar-H), 5.04 (m, 2H, NC*Hi*Pr), 1.79 (d, J = 6.7 Hz, 12H, C*H$_3$i*Pr).
^{13}C (^{1}H) NMR (101 MHz, $CDCl_3$): δ 139.14 (s, N_2*C*H), 131.13 (s, C_q), 127.25 (s, C_{Ar}), 114.01 (s, C_{Ar}), 52.46 (s, *C*H*i*Pr), δ 22.91 (s, *C*H$_3$*i*Pr).
^{19}F NMR (376, $CDCl_3$): δ -78.23
Elemental analysis not performed due to issues with HF and our instrument

5-((12-(4-(Ferrocenyl)-1*H*-1,2,3-triazol-1-yl)dodecyl)oxy)-1,3-diisopropyl-1*H*-benzo[*d*]imidazol-3-ium iodide (1c)

Prepared via the method reported in reference ([14]).

5-((12-(4-(Ferrocenyl)-1*H*-1,2,3-triazol-1-yl)dodecyl)oxy)-1,3-diisopropyl-1*H*-benzo[*d*]imidazol-3-ium hydrogen carbonate (3c)

Method 2: Hydrogen carbonate-anion exchange resin

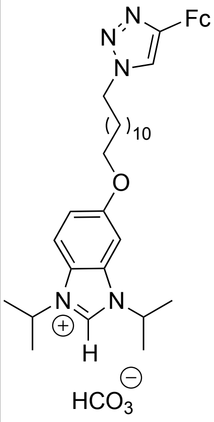

(3c)

Resin-HCO_3 (0.3 mL, 3 equiv.) suspended in water was measured in a graduated cylinder and transferred to 20 mL vial where the resin was allowed to settle and water was decanted off. The resin was washed with methanol (3 x 1mL). 5-(12-(4(Ferrocenyl)-1H-1,2,3-triazol-1-yl)dodecyloxy)-1,3-diisopropyl-1H-benzo[d]imidazol-3-ium iodide (**1c**) (56 mg, 0.07 mmol) as dissolved in 5 mL methanol and transferred to the resin. The mixture was stirred for 30 min. The red solution was passed through a cotton plug. Solvent was evaporated using a rotavap at 40 °C then *in vacuo* for 2 h to give an orange solid (35 mg, 71%).

^{1}H NMR (400 MHz, CD_3OD): δ 8.01 (s, 1H, triazole), 7.88 (d, 1H, J_{HH} = 9.0 Hz, Ar*H*), 7.42 (s, 1H, Ar*H*), 7.28 (d, 1H, J_{HH} = 8.6 Hz, Ar*H*), 5.00 (m, 2H, C*H*-$(CH_3)_3$), 4.73 (s, 2H, ferrocene), 4.40 (t, 2H, J_{HH} = 6.8 Hz, ferrocene), 4.30 (m, 2H, N-C*H$_2$*), 4.11(t, J_{HH} = 6.4 Hz, O-C*H$_2$*), 4.02 (m, 5H, ferrocene), 1.93 (m, 2H), 1.82 (m, 2H), 1.70 (m, 12H, -C*H$_2$*-), 1.49 (m, 2H), 1.31 (m, br, 16H).
^{13}C (^{1}H) NMR (CD_3OD): δ 161.35 (s, H*C*O_3^-), 160.39 (s, *C*q), 147.94 (s, *C*q), 139.39 (s, N-*C*H=N), 133.80 (s, C_q), 126.52 (s, C_q), 121.36 (s, triazole), 118.69 (s, Ar), 115.63 (s, Ar), 97.46 (s, Ar), 76.19 (s, Ar),70.53 (s, ferrocene), 70.24 (s, *C*H_2-O), 69.77 (s, ferrocene), 67.60 (s, ferrocene), 52.80 (s, CH_3-*C*H-CH_3), 52.24 (s, CH_3-*C*H-CH_3), 51.32 (s, *C*H_2-triazole), 31.16 (s), 30.60 (s), 30.56 (s), 30.48 (s), 30.46 (s), 30.38 (s), 30.18 (s), 29.95 (s), 27.79 (s), 27.09 (s), 22.17 (s, *C*H_3), 22.11 (s, *C*H_3).
ATR-IR: strong peaks for CO_2 asym. str. at 1661 cm^{-1} and sym. str. at 1288 cm^{-1}
HRESI-MS (m/z) for $C_{37}H_{52}FeN_5O^+$ [M-HCO_3]$^+$: 638.3491, Calc.: 638.3516.

(3.6) 5-(Dodecyloxy)-1,3-diisopropyl-1H-benzo[d]imidazol-3-ium iodide (1d)

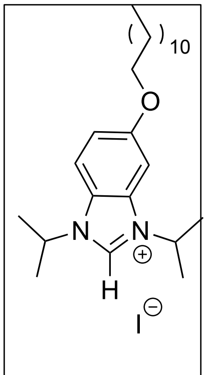

(1d)

5-(Dodecyloxy)-1,3-diisopropyl-1H-benzo[d]imidazol-3-ium iodide (**1d**) was prepared using a similar method as described in the literature procedures [14] with a slight modification where 2-iodopropane (1.6 mL, 16 mmol, 5 equiv.) was slowly added to a suspension of 5-(dodecyloxy)-1*H*-benzo[*d*]imidazole (971 mg, 3.2 mmol) and Cs_2CO_3 (1.04 g, 3.2 mmol) in acetonitrile (16 mL). The mixture was heated to 90°C in a two-necked round bottom flask under an argon atmosphere for 48 h. The reaction mixture was allowed to cool to room temperature. Water (20 mL) was added to the reaction mixture. The reaction mixture was then extracted with dichloromethane (3 x 30 mL). The combined organic layers were dried over anhydrous magnesium sulfate, filtered and then concentrated *in vacuo*. The crude solid was triturated and sonicated in diethyl ether (3 × 6 mL). Subsequent drying under high vacuum afforded the desired product as an off-white powder (1.30 g, 78% yield).

^{1}H NMR (400 MHz, $CDCl_3$) δ 10.81 (s, 1H, N-C*H*=N), 7.64 (d, 1H, J_{HH} = 9.2 Hz, Ar*H*), 7.21 (dd, 1H, J_{HH} = 9.2, 2.0 Hz, Ar*H*), 7.09 (d, 1H, J_{HH}= 1.9 Hz, Ar*H*), 5.10 (m, 2H, C*H*-$(CH_3)_3$), 4.05 (t, 2H, J_{HH} = 6.4 Hz, O-CH_2), 1.84 (m, 14H), 1.48 (m, 2H), 1.26 (m, 16H), 0.87 (t, 3H, J_{HH} = 6.6 Hz). Spectra were consistent with literature reports.(*11*)

5-(Dodecyloxy)-1,3-diisopropyl-1*H*-benzo[*d*]imidazol-3-ium hydrogen carbonate (3d)

Method 2: Hydrogen carbonate-anion exchange resin

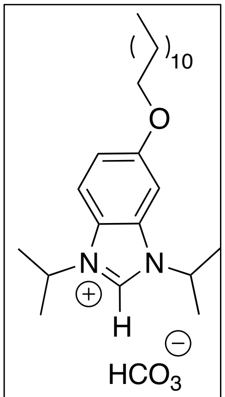

(3d)

Resin-HCO_3 (9.4 mL, 10 equiv.) suspended in water was measured in a graduated cylinder and then transferred to 50 mL round bottom flask, allowed to settle and water was decanted.5-(Dodecyloxy)-1,3-diisopropyl-1*H*-benzo[*d*]imidazol-3-ium iodide (**1d**, 358.5 mg, 0.75 mmol) was dissolved in 7.5 mL acetonitrile and transferred to the resin suspension. Water, (7.5 mL) was added to the resin. The mixture was stirred for 30 min. The bicarbonate solution was passed through a cotton plug to remove any resin beads and the resin was washed with (3 x 2 mL 1:1 water : acetonitrile). Solvents were removed overnight under a stream of air. The resulting yellow oily solid was triturated and sonicated in 10 % acetone/diethyl ether (3 × 4 mL), which was then decanted off. Subsequent drying under vacuum afforded the desired product as an off-white powder (206 mg, 61% yield). Mp. 68-71°C (dec.)

^{1}H NMR (400 MHz, CD_3OD): δ 7.89 (d, *J*= 9.2 Hz, 1H, CH_{Ar}), 7.44 (d, *J*= 2.1 Hz, 1H,CH_{Ar}), 7.30 (dd, *J* = 9.2, 2.2 Hz, 1H, CH_{Ar}), 5.10 – 4.92 (m, 2H, C*H*-$(CH_3)_2$), 4.13 (t, *J* = 6.4 Hz, 2H,-O-CH_2), 1.85 (m, 2H), 1.71 (m, 12H, CH-$(CH_3)_2$), 1.53 (s, 2H), 1.29 (s, 16H), 0.90 (t, J_{HH} = 6.8 Hz, 3H, CH_3). The N_2C*H* and *H*CO_3 protons could not be observed due to their rapid exchange with the deuterated solvent on the NMR time scale.
^{13}C (^{1}H) NMR (CD_3OD): δ 161.43 (HCO_3^-),160.43 (C_q-O-CH_2), 137.96 (C_{Ar}, N_2*C*H), 133.83 (C_q), 126.54 (C_q), 118.71 (C_{Ar}), 115.62 (C_{Ar}), 97.41 (C_{Ar}), 70.23

(CH_2-O), 52.80 ($CH(CH_3)_2$), 52.23 ($CH(CH_3)_2$), 33.07 (CH_2), 30.76 (CH_2), 30.75 (CH_2), 30.70 (CH_2), 30.47 (CH_2), 30.23 (CH_2), 27.15 (CH_2), 23.73 (CH_2), 22.17 ($CH(CH_3)_2$), 22.11($CH(CH_3)_2$), 14.43 (CH_3)
ATR-IR: strong peaks for CO_2 asym. str. at 1620 cm^1 and sym. str. at 1371 cm^1 .
Anal. Calc. for $C_{26}H_{44}N_2O_4$: C, 69.61; H, 9.89; N, 6.24. Found: C, 68.99; H, 9.76; N, 6.30.
HRESI-MS (m/z) for $C_{25}H_{43}N_2O^+$ $[M-HCO_3]^+$: 387.3370, Calc.: 387.3370.

Method 3: Hydrogen carbonate-anion exchange resin with triflate starting material

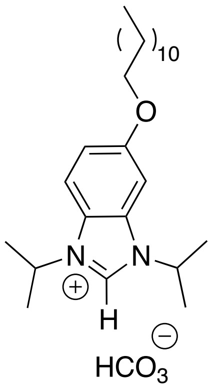
(3d)

Resin-HCO_3 (1.5 mL, 3 equiv.) suspended in water was measured in a graduated cylinder and transferred to 20 mL vial where the resin was allowed to settle and water was decanted off. The resin was washed with methanol (3 x 2 mL). 5-(dodecyloxy)-1,3-diisopropyl-1*H*-benzo[*d*]imidazol-3-ium trifluoromethanesulfonate (215 mg, 0.4 mmol) was dissolved in 2 mL methanol and transferred to the resin. The mixture was stirred for 15 min. The solution was passed through a cotton plug. Solvent was evaporated under vacuum and the crude oily product was triturated and sonicated in hexane (2 x 4mL). Subsequent drying under high vacuum afforded the desired product as an off-white powder (153 mg, 85% yield).
Spectra matched those shown above. Anal. Calc. for $C_{26}H_{44}N_2O_4$: C, 69.61; H, 9.89; N, 6.24. Found: C, 68.98; H, 10.10; N, 6.10.

1-isopropyl-5-propoxy-1*H*-benzo[*d*]imidazole and 1-isopropyl-6-propoxy-1*H*-benzo[*d*]imidazole

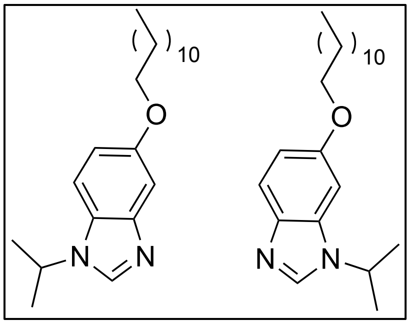

A mixture of 5-propoxy-1H-benzo[*d*]imidazole (0.917 g, 3.03 mmol), anhydrous Cs_2CO_3 (1.480 g, 4.55 mmol) and 2-bromopropane (0.854 mL, 9.09 mmol) in acetonitrile (21 mL) was stirred at 90°C in a two-neck.round bottomed flask attached to a reflux condenser under an argon atmosphere for 14 h. After cooling to RT, the resulting mixture was filtered by suction and washed with dichloromethane (ca. 30 mL), then solvents were removed. The crude mixture was purified by silica column chromatography using hexane-ethyl acetate 1:2 mixture (R_f = 0.19) yielding a yellow liquid (0.673 g, 64%) of the products as a 1:1 mixture of the two regioisomers.
^{1}H NMR (400 MHz, $CDCl_3$) δ 7.91, 7.87 (s, 2H, N-C*H*=N), 7.67 (d, *J*= 9.2 Hz, 1H, CH_{Ar}), 7.28 (m, 2H, CH_{Ar}), 6.90 (m, 3H, CH_{Ar}), 4.54 (m, 2H, C*H*-$(CH_3)_2$), 4.00 (t, *J* = 6.4 Hz, 4H,-O-CH_2), 1.81 (m, 4H), 1.58 (m, 12H), 1.48 (m, 4H), 1.27 (m, 32H), 0.88 (t, J_{HH} = 6.6 Hz, 6H, CH_3).
^{13}C (^{1}H) NMR (101 MHz, $CDCl_3$): δ 156.03, 155.51, 144.84, 140.23, 139.30, 138.52, 133.89, 127.82, 120.70, 113.40, 111.67, 110.34, 103.31, 94.61, 77.16, 68.81, 68.68, 47.74, 47.44, 31.92, 29.67, 29.64, 29.61, 29.44, 29.35, 26.12, 22.69, 22.57, 22.47, 14.11.
EI-MS (m/z) for $C_{22}H_{36}N_2O$: 344.2813, Calc.: 344.2828.

5-(dodecyloxy)-1,3-diisopropyl-1*H*-benzo[*d*]imidazol-3-ium trifluoromethanesulfonate

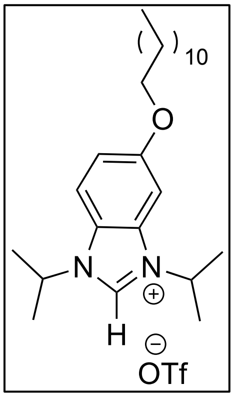

In an oven-dried schlenck flask, triflic anhydride (0.50 mL, 3.0 mmol) was slowly added to a stirred solution of anhydrous 2-propanol (0.23 mL, 3.0 mmol) and anhydrous pyridine (0.24 mL, 3.0 mmol) in dry dichloromethane (5 mL) at −20°C under argon resulting in the formation of white precipitate. The reaction mixture was stirred at −20°C for 30 minutes and then at RT for additional 30 minutes. Dry pentane (5 mL) was added to further precipitate the formed salts and the reaction mixture was then filtered through a pad of Celite directly to a solution of 1-isopropyl-5-propoxy-1*H*-benzo[*d*]imidazole and 1-isopropyl-6-propoxy-1*H*-benzo[*d*]imidazole (0.344 g, 1.0 mmol) in dry dichloromethane (5 mL). After stirring for 10 min at −10°C and then at RT for 14 h under argon, the reaction mixture was quenched with a saturated solution of $NaHCO_3$ (15 mL). The layers were separated, and the aqueous layer was extracted twice with dichloromethane (2 x 15 mL). The combined organic layer was dried over $MgSO_4$, filtered and concentrated *in vacuo*. The residue was triturated and sonicated in diethyl ether (2 × 5 mL), which was then decanted off. Subsequent drying under vacuum afforded the desired product as an off-white powder (0.537 g, 82% yield).

^{1}H NMR (400 MHz, $CDCl_3$): δ 9.75 (s, 1H, N-C*H*=N), 7.63 (d, 1H, J_{HH} = 9.2 Hz), 7.22 (dd, 1H, J_{HH} = 9.2, 2.0 Hz, Ar*H*), 7.09 (s, 1H), 4.95 (m, 2H, C*H*-$(CH_3)_3$), 4.05 (t, 2H, J_{HH} = 6.5 Hz, -O-CH_2-), 1.85 (m, 2H), 1.77 (m, 12H), 1.49 (m, 2H), 1.26 (m, 16H), 0.87 (t, 3H, *J*HH = 6.8 Hz).

^{13}C (1H) NMR ($CDCl_3$): δ 158.88 (C_q, C_{Ar}-O-CH_2-), 137.95 (C_{Ar}, N=*C*H-NH), 132.25 (C_q), 124.92 (C_q), 117.45 (C_{Ar}), 114.41 (C_{Ar}), 96.53 (C_{Ar}), 69.30 (-*C*H_2-O-), 52.29 (*C*H-$(CH_3)_2$), 51.80 (*C*H-$(CH_3)_2$), 31.91, 29.66, 29.63, 29.59, 29.55, 29.36, 29.34, 29.04, 25.98, 22.68, 21.85 (CH-(CH_3)$_2$), 21.72 (CH-(CH_3)$_2$), 14.10 (*C*H_3).

^{19}F NMR (376, $CDCl_3$): δ -78.71

HRESI-MS (m/z) for $C_{25}H_{43}N_2O^+$ [M-CF_3O_3S]$^+$: 387.3371, Calc.: 387.3370.

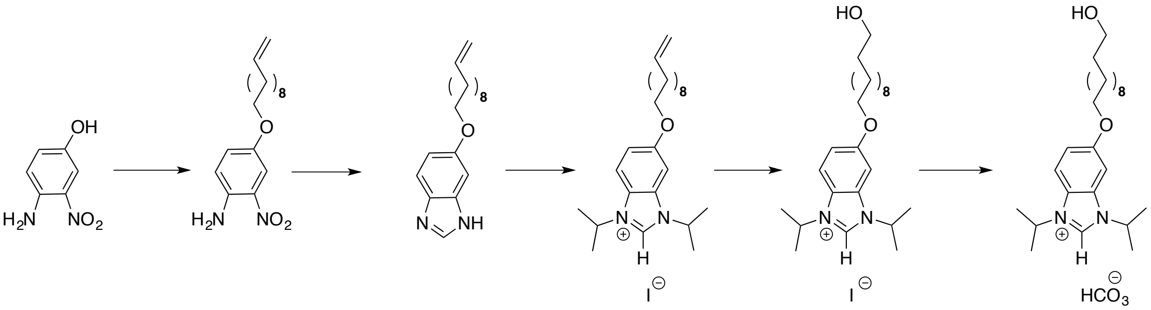

2-nitro-4-(undec-10-en-1-yloxy)aniline

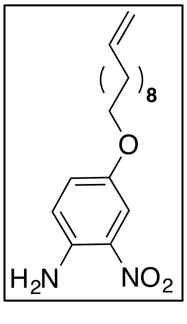

Modified from a previously reported preparation.[14] To a solution of 4-amino-3-nitrophenol (1.000 g, 6.5 mmol) and 11-bromo-1-undecne (1.644 g, 1.55 mL, 7.1 mmol) in anhydrous acetonitrile (50 mL), potassium carbonate (0.986 g, 7.1 mmol) was added. The mixture was stirred at 80°C for 17 h under argon. Then the solvent was evaporated and the residue was dissolved in dichloromethane and filtered. Dichloromethane was evaporated and the crude product was separated by flash-chromatograph

using hexane-ethyl acetate gradient mixtures. (2.444 g, 85% yield).

^{1}H NMR (400 MHz, $CDCl_3$): δ 7.53 (d, 1H, J = 2.8 Hz), 7.06 (dd, 1H, J = 9.1, 2.8 Hz), 6.75 (d, 1H, J = 9.1 Hz), 5.88 (s, 2H, N*H₂*), 5.81 (m, 1H, -C*H*=CH_2), 4.96 (m, 2H, -CH=C*H₂*), 3.91 (t, 2H, J = 6.5 Hz, -O-C*H₂*-), 2.04 (m , 2H, -C*H₂*-C*H*=CH_2), 1.76 (m, 2H,-C*H₂*-CH_2O-) 1.45-1.26 (m, 12H).
^{13}C (^{1}H) NMR (101 MHz, $CDCl_3$): δ 150.41 (s, C_q, *C*-O-CH_2-), 139.93 (s, C_q, *C*-NO_2), 139.34 (s, -*C*H=CH_2), 131.66 (s, C_q, *C*-NH_2), 127.24 (s, Ar), 120.13 (s, Ar), 114.25 (s, -CH=*C*H_2), 107.24 (s, Ar), 68.91 (s, -*C*H_2-O), 33.92 (s), 29.61 (s), 29.52 (s), 29.46 (s), 29.23 (s), 29.04 (s), 26.10 (s).
Anal. Calc. for $C_{17}H_{26}N_2O_3$: C, 66.64; H, 8.55; N, 9.14. Found: C, 66.68; H, 8.58; N, 9.10.
TOF MS (m/z) for $C_{17}H_{26}N_2O_3$: 306.1929, Calc.: 306.1943.

5-(undec-10-en-1-yloxy)-1*H*-benzo[*d*]imidazole

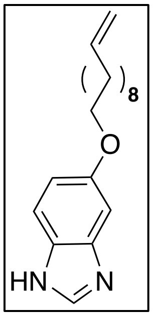

Modified from a previously reported preparation.[14] Formic acid (25 mL) was added to a mixture of 2-nitro-4-(undec-10-en-1-yloxy)aniline (1.532 g, 5 mmol), iron powder (2.790 g, 50 mmol), and ammonium chloride (2.670 g, 50 mmol) in isopropyl alcohol (50 mL). The resulting mixture was stirred at 80°C for 3.5 h, then cooled to room temperature and filtered through sintered glass filter. The solid was washed with isopropyl alcohol (3 x 5 mL). The filtrate was evaporated to dryness and 30 mL of saturated sodium bicarbonate solution was added carefully to avoid significant foaming. Then sodium bicarbonate (powder) was added portion-wise until pH 6 was achieved. Then the suspension was extracted with dichloromethane (5 x 30 mL). The combined organic layers were dried over anhydrous magnesium sulfate, filtered and evaporated to give the product. (1.407 g, 98% yield)

^{1}H NMR (500 MHz, $CDCl_3$) δ 7.97 (s, N=C*H*-NH, 1H), 7.53 (d, 1H, J = 8.7 Hz), 7.07 (s, 1H), 6.93 (d, 1H, J = 8.4 Hz), 5.81 (m, 1H, -C*H*=CH_2), 4.96 (m, 2H, -CH=C*H₂*), 3.97 (t, 2H, J = 6.4 Hz, -O-C*H₂*-), 2.04 (m, 2H, -C*H₂*-CH=CH_2), 1.79 (m, 2H, -C*H₂*-CH_2O-), 1.46 (m, 2H), 1.33 (m, 12H).
^{13}C (^{1}H) NMR (126 MHz, $CDCl_3$): δ 156.39 (s, C_q, C_{Ar}-O-CH_2-), 140.13 (s, C_{Ar}, N=*C*H-NH), 139.37 (s, -*C*H=CH_2),132.76 (s, C_{Ar}), 116.64 (s, C_{Ar}), 114.26 (s, -CH=*C*H_2), 113.40 (s, C_{Ar}), 98.43 (s, C_{Ar}), 68.88 (s, -*C*H_2-O-), 33.94 (s), 29.84 (s), 29.66 (s), 29.57 (s), 29.55 (s), 29.48 (s), 29.26 (s), 29.07 (s), 26.24 (s).
TOF MS (m/z) for $C_{18}H_{26}N_2O$: 286.2045, Calc.: 286.2056.

1,3-diisopropyl-5-(undec-10-en-1-yloxy)-1*H*-benzo[*d*]imidazol-3-ium iodide

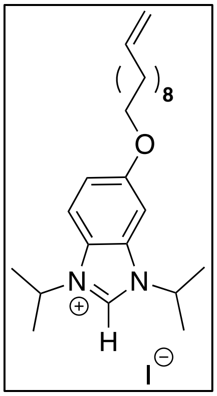

Modified from a previously reported preparation.[14] To a suspension of 5-(undec-10-en-1-yloxy)-1*H*-benzo[*d*]imidazole (286.4 mg, 1 mmol) and Cs_2CO_3 (325.8 mg, 1.1 mmol) in acetonitrile (5 mL), 2-iodopropane (499 µL, 5 mmol) was slowly added. The mixture was stirred at 90 °C in a sealed pressure tube under an argon atmosphere for 48 h. The reaction mixture was allowed to cool to room temperature. Water (20 mL) was added to the reaction mixture which was then extracted with dichloromethane (3 x 15 mL). The combined organic layers were dried over anhydrous magnesium sulfate, filtered and then concentrated *in vacuo*. The crude solid was triturated and sonicated in diethyl ether (5 × 5 mL). Subsequent drying under high vacuum afforded the desired product as a yellow powder (411 mg, 82% yield).

^{1}H NMR (500 MHz, $CDCl_3$): δ 10.55 (s, 1H), 7.66 (d,1H, *J* = 9.2 Hz), 7.21 (dd, 1H, *J* = 9.2, 2.2 Hz), 7.12 (d, 1H, *J* = 2.1 Hz), 5.79 (m, 1H, -C*H*=CH_2), 5.13 (m, 2H, 2H, C*H*-$(CH_3)_2$), 4.94 (m, 2H, -CH=CH_2), 4.06 (t, 2H, *J* = 6.4 Hz, -O-CH_2-), 2.02 (m , 2H, -CH_2-CH=CH_2), 1.83 (m, 14H, -CH_2-CH_2O-, CH-(CH_3)$_2$),1.47 (m, 2H), 1.32 (m, 10H).

^{13}C (^{1}H) NMR (126 MHz, $CDCl_3$) δ 158.94 (s, C_q, C_{Ar}-O-CH_2-), 139.24 (s, -*C*H=CH_2), 138.43 (s, C_{Ar}, N=*C*H-NH), 132.19 (s, C_q), 124.87 (s, C_q), 117.53 (s, C_{Ar}), 114.65 (s, C_{Ar}), 114.23 (s, -CH=*C*H_2), 96.79 (s, C_{Ar}), 69.50 (s, -*C*H_2-O-), 52.53 (s, *C*H-$(CH_3)_2$), 52.10 (s, *C*H-$(CH_3)_2$), 33.86 (s,-*C*H_2-CH=CH_2), 29.55 (s), 29.48 (s), 29.42 (s), 29.17 (s), 29.12 (s), 28.98 (s), 26.07 (s), 22.46 (s, CH-(CH_3)$_2$), 22.39 (s, CH-(CH_3)$_2$).

HRMS (ESI): $[M - I]^+$. HRMS: calcd. for $C_{24}H_{39}N_2O$ $[M - I]^+$ 371.3057; found 371.3051.

5-((11-hydroxyundecyl)oxy)-1,3-diisopropyl-1*H*-benzo[*d*]imidazol-3-ium iodide

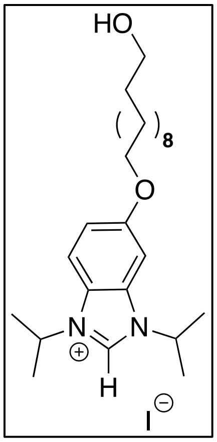

Modified from a previously reported preparation.[15] In an oven-dried shlenck flask, 1,3-diisopropyl-5-(undec-10-en-1-yloxy)-1*H*-benzo[*d*]imidazol-3-ium iodide **(**249.24 mg, 0.5 mmol**)** was dissolved in 2.4 mL dry THF and the solution was cooled to 0 °C (ice bath). Hydroboration was initiated by dropwise addition of a 1.0 M solution of borane-THF (1.6 mL, 1.6 mmol), followed by stirring the mixture at room temperature for 2 h that changed gradually from a yellow mixture to a red solution. Cold water (4 mL) was added dropwise and then sodium perborate (249.52 mg, 2.5 mmol) was introduced. The mixture was vigorously stirred at room temperature for 2h after which it formed a yellow suspension. Water (20 mL) was added to the reaction mixture, which was then extracted with dichloromethane (3 x 20 mL). The combined organic layers were dried over anhydrous magnesium sulfate, filtered and then concentrated. The crude oily product was triturated and sonicated in diethyl ether (3 × 4mL). Subsequent drying under high vacuum afforded a yellow powder (202.6 mg, 78.4% yield).

^{1}H NMR (400 MHz, $CDCl_3$ + D_2O): δ 10.73 (s, 1H), 7.62 (d,1H, *J* = 9.2 Hz), 7.21 (dd, 1H, *J* = 9.3, 1.8 Hz), 7.08 (d, 1H, *J* = 1.6 Hz), 5.12 (m, 2H, C*H*-$(CH_3)_2$), 4.05 (t, 2H, *J* = 6.4 Hz, -O-CH_2-), 3.66 (t, 2H, *J* = 6.6 Hz, -CH_2-OH), 1.85 (m, 14H, -CH_2-CH_2O-, CH-(CH_3)$_2$), 1.57 (m, 2H, CH_2-CH_2O*H*, after D_2O-exchange), 1.49 (m, 2H), 1.30 (m, 12H).

^{13}C (^{1}H) NMR (126 MHz, $CDCl_3$): δ 158.90 (s, C_q, C_{Ar}-O-CH_2-), 138.40 (s, C_{Ar}, N=*C*H-NH), 132.15 (s, C_q), 124.83 (s, C_q), 117.50 (s, C_{Ar}), 114.65 (s, C_{Ar}), 96.80 (s, C_{Ar}), 69.47 (s, -*C*H_2-O-), 62.92 (s, -*C*H_2OH), 52.45 (s, *C*H-$(CH_3)_2$), 52.02 (s, *C*H-$(CH_3)_2$), 32.80 (-*C*H_2-CH_2OH), 29.55 (s), 29.57 (s), 29.50 (s), 29.47 (s), 29.43 (s), 29.32 (s), 29.06 (s), 25.99 (s), 25.79 (s), 22.40 (s, CH-(CH_3)$_2$), 22.34(s, CH-(CH_3)$_2$).

Anal. Calc. for $C_{24}H_{41}N_2O_2I$: C, 55.81; H, 8.00; N, 5.42. Found: C, 55.28; H, 8.00; N, 5.50.

HRMS (ESI): $[M - I]^+$. HRMS: calcd. for $C_{24}H_{41}N_2O_2$ $[M - I]^+$ 389.3163; found 389.3159.

5-((11-hydroxyundecyl)oxy)-1,3-diisopropyl-1H-benzo[d]imidazol-3-ium hydrogen carbonate

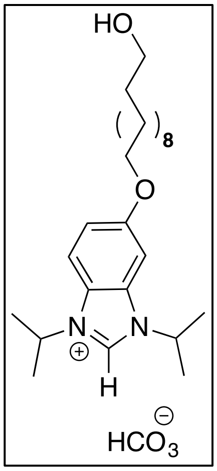

Resin-HCO_3 (1.2 mL, 3 equiv.) suspended in water was measured in a graduated cylinder and transferred to 20 mL vial where the resin was allowed to settle and water was decanted off. The resin was washed with methanol (3 x 2 mL). 5-((11-hydroxyundecyl)oxy)-1,3-diisopropyl-1H-benzo[d]imidazol-3-ium iodide (154.9 mg, 0.3 mmol) was dissolved in 3 mL methanol and transferred to the resin. The mixture was stirred for 15 min. The solution was passed through a cotton plug. Solvent was evaporated and the crude oily product was triturated and sonicated in diethyl ether (4mL). Subsequent drying under high vacuum afforded the primary alcohol as an off-white powder (95 mg, 70% yield).

^{1}H NMR (500 MHz, CD_3OD): δ 7.90 (d, J = 9.2 Hz, 1H), 7.45 (d, 1H, J = 1.8 Hz), 7.31 (dd, J = 9.2, 2.1 Hz, 1H), 5.02 (m, 2H, C*H*-$(CH_3)_2$), 4.14 (t, 2H, J = 6.3 Hz, -OC*H₂*-), 3.54 (t, 2H, J = 6.6 Hz, -C*H₂*OH), 1.86 (m, 2H, -C*H₂*-CH_2O-), 1.72 (m, 12H, CH-$(CH_3)_2$), 1.53 (m, 4H), 1.34 (m, 12H). The N_2C*H* and *H*CO_3 protons could not be observed due to their rapid exchange with the deuterated solvent on the NMR time scale. ATR-IR: strong peaks for CO_2 asym. str. at 1656 cm^1 and sym. str. at 1342 cm^1 .

^{13}C (^{1}H) NMR (126 MHz, $CDCl_3$): δ 161.41 (s, HCO_3^-),160.42 (s, C_{Ar}-O-CH_2-), 137.84 (s, C_{Ar}, N=*C*H-NH), 133.83 (s, C_q), 126.54 (s, C_q), 118.70 (s, C_{Ar}), 115.64 (s, C_{Ar}), 97.41 (s, C_{Ar}), 70.22 (s, -*C*H_2-O-), 62.98 (s, -*C*H_2OH), 52.80 (s, *C*H-$(CH_3)_2$), 52.23 (s, *C*H-$(CH_3)_2$), 33.66 (-*C*H_2-CH_2OH), 30.72 (s), 30.68 (s), 30.64 (s), 30.59 (s), 30.46 (s), 30.22 (s), 27.14 (s), 26.95 (s), 22.16 (s, CH-$(CH_3)_2$), 22.11(s, CH-$(CH_3)_2$).

Anal. Calc. for $C_{25}H_{42}N_2O_5$: C, 66.64; H, 9.39; N, 6.22. Found: C, 68.63; H, 9.79; N, 6.65.

HRMS (ESI): $[M - HCO_3]^+$. HRMS: calcd. for $C_{24}H_{41}N_2O_2^+$ $[M - HCO_3]^+$ 389.3163; found 389.3163.

Deposition of Carbenes on Gold Surfaces

Cleaning of gold surfaces

Vapor deposition experiments were conducted in three separate stainless steel UHV chambers hosting an Ar ion sputtering gun and annealing facilities for sample cleaning. TPD data were collected in a UHV chamber (base pressure P= $1x10^{-9}$ mbar) equipped with a quadrupole mass spectrometer (SPECTRA, Microvision Plus) in direct line-of-sight with the crystal, and a LEED/Auger spectrometer (SpectaLEED, Omicron). HREELS experiments were conducted in a second chamber (base pressure = $5x10^{-11}$ mbar) hosting a double pass HREEL spectrometer (Ibach, HIB1000) separated from an adjacent preparation chamber. STM images were recorded in a third chamber (base pressure =$1x10^{-10}$ mbar) equipped with a variable temperature scanning tunneling microscope (Omicron), and LEED optics. An electrochemically etched W tip was used for STM imaging typically employing bias voltages of ±0.6 V and tunneling currents of 300 pA. Image processing and filtering have been applied to the STM data using WSxM.[16]

An Au (111) single crystal was cleaned by 10 cycles of annealing at 775 K and Ar ion sputtering (1.25 kV). Each cleaning cycle was terminated by annealing to 775 K for 15 minutes before the crystal was cooled down to room temperature. Annealing was done by direct or resistive heating of the sample, and the temperature monitored by means of a type-K thermocouple. Cleanliness of the sample was assessed either by monitoring the TPD traces during the final annealing cycle until no desorbing species were observed, or by HREELS until a featureless spectrum was obtained, or by STM and LEED until the $22\times\sqrt{3}$ reconstruction[17] was visible.

Au(111) on mica

Prior to functionalization the Au(111) films were cleaned by washing the films in 3 x 2 mL of methanol, drying them under an argon gas (4.8 Praxair) stream for 1 minute, then cleaning them with plasma generated from room air at a medium RF level and a pressure kept between 300 and 500 mtorr for 1 minute. The films were then used immediately for functionalization.

Au/Si for Electrochemical Cycling

NHC films prepared for electrochemical cycling with **3d** using Au/Si substrates, which were prepared by electron-beam deposition at a thickness of 200 nm Au on a Si wafer with 20 nm of Ti as the adhesion layer (Nanofabrication Facility at University of Western Ontario).

Au microelectrodes: synthesis and cleaning

Homemade gold microelectrodes were used for electrochemistry using NHC derivative **3c**. The microelectrodes were manufactured by sealing gold wire with diameter of 25 mm into a glass capillary. After this, the microelectrodes were polished with 3- micron lapping films to expose the cross-section of Au wires and then the Au surfaces was polished down with 0.3-micron and 0.05-micron Al_2O_3 lapping films. The microelectrodes were sonicated in deionized water, ethanol and deionized water for 20 minutes respectively. The microelectrodes were electrochemically cleaned prior to each experiment by running a cyclic voltammetry immersed in a 0.5 M KOH solution between -2 V and 0 V for 100 cycles, followed by 0.5 M H_2SO_4 solutions between 0 V and +1.4 V for 100 cycles at a scan rate of 0.5 $V{\cdot}s^{-1}$. The electrodes were always rinsed with deionized water thoroughly between steps.

Preparation of SPR Biosensing Chip Surfaces

Gold sensor surfaces (SIA kit Au, GE Healthcare) were stored at 4 °C. Prior to use, initial cleaning of these sensor surfaces was performed by immersion in a mixture of NH_4OH: H_2O_2: H_2O (1:1:5) at 80 °C for 0.5 h, rinsed thoroughly with milliQ-water, and drying under a stream of nitrogen. Additional cleaning was performed at 60 W for 15 min with a Harrick Plasma Cleaner/Sterilizer (PDG-32G), followed by immediately immersing the gold sensor surfaces into a 10 mM solution of NHC molecules dissolved in dry methanol (48 h, room temperature, without light). The sensor surfaces were then rinsed with approximately 20 mL of methanol followed by approximately 100 mL of milliQ-water to remove excess absorbate, dried under nitrogen, and then mounted on a support for insertion into the cartridge. Special care was taken to

prevent artificial scratches or impurities from depositing onto the hydrophobic NHC sensor surfaces.

Preparation of SPR Biosensing Chip Surfaces with Dextran on tether

The following procedure is modified from Granqvist et al.[18] and Lofas and Johnsson.[19] The sensor chips (Au/glass Biacore chips) were first cleaned by keeping them in a boiling H_2O_2 (30%):NH_3:H_2O (1:1:5) solution for 10 min, washed thoroughly with ultrapure water, washed with methanol, dried under an argon gas for 1min, then cleaned with plasma generated from room air at a medium RF level and pressure kept between 300 and 500 mtorr for 10 minute. The sensors were immersed in a solution of 10 mM 5-((11-hydroxyundecyl)oxy)-1,3-diisopropyl-1*H*-benzo[*d*]imidazol-3-ium hydrogen carbonate in a methanol solution (2 mL over each chip) for 24 h, after which they were washed thoroughly with approximately 20 mL of methanol followed by approximately 100 mL of milliQ-water. The chips were then allowed to react for 3 h with epichlorohydrin (2% v/v) in 0.1 M NaOH, rinsed with plenty of water, transferred to a 300 g/L solution of dextran in 0.1 M NaOH, and left to react for 24 h. The sensors were washed thoroughly with ultrapure water and immersed in 1.0 M bromoacetic acid in 2 M NaOH for 24 h, after which they were thoroughly washed with ultrapure water, dried with an argon stream, assembled on the Biacore stand and stored under argons in plastic tube.

Preparation of SAMs

Hydrogen Carbonate Salt Method

Self-assembled monolayers were prepared by immersion of Au(111) on mica substrates in 1 to 10mM solutions of the corresponding benzimidazolium hydrogen carbonate salt in methanol for 24 hours at room temperature in air. Substrates then were rinsed in methanol (5 x 2 mL) and dried under an argon gas (4.8 Praxair) stream for 1 minute.

Free Carbene Method

Self-assembled monolayers were prepared by immersion of Au(111) on mica substrates in a 1 mM solution of the corresponding free carbene [14] dissolved in dry toluene at room temperature in the glove box. Substrates were then rinsed in toluene (10 x 2 mL) and dried under an argon gas (4.8 Praxair) stream for 1 minute.

Vapor Deposition Method

iPr_2bimy(H)[HCO_3] (**3a**) was outgassed for 12 h at room temperature on a differentially pumped manifold separated from the UHV system by a gate valve. Dosing was achieved by resistively heating a glass microcapillary wrapped in Ta wire containing compound **3a** [**Supplementary Figure 2**]. Calibration of dosing temperature was achieved by gradually increasing the temperature until the mass spectrum displayed a fragmentation pattern consistent with the presence of the molecule in the gas phase. Temperature programmed desorption experiments were conducted for various exposure times at a heating rate of $\beta = 3.5\ K \cdot s^{-1}$. Coverages are reported as fractions of a full monolayer. One monolayer (1 ML) is defined as the total integrated area of the peak for the fragment m/z=41 amu

(most intense) achieved upon saturation exposure (adsorption beyond a chemisorbed monolayer was not observed at 300 K). Subsequent coverages are expressed as the area under the curve relative to the saturated peak area. The best fit accompanying the data was achieved by the sum of Gaussian functions and a 10-15 point baseline. HREELS spectra were collected in specular mode (θ_i=θ_f=45°) at 20 scans per spectrum, with a primary beam energy of E_0= 5 eV and at a resolution of 3.85 meV measured as the FWHM of the elastic peak. All spectra have been normalised to the elastic peak.

iPr_2bimy(H)[HCO_3] (**3a**) was dosed on the Au (111) crystal, which was held at room temperature. Multiplexing between 1 and 150 amu in TPD experiments revealed fragments m/z= 2, 27, 39 and 41 amu desorbing from the surface. No other signal was detected that could indicate co-adsorption of the carbonate counterion (m/z= 60, 44, or 28 amu) nor any other higher mass fragment.

Deposition on electrodes

Au/Si substrates and ultramicroelectrodes were modified by immersion in 1 mM solutions of the desired NHC derivative hydrogen carbonate salt in dry methanol for 24 h at room temperature. The substrates and electrodes were rinsed in methanol (10 x 2 mL) and dried under an argon gas (4.8 Praxair) stream.

Chemical Stability Tests

All films were prepared using the hydrogen carbonate salt **3a** and the solution deposition method previously described. Data for stability measurements are given in Chemical Stability Results.

pH Stability Tests

NHC films derived from *i*Pr_2bimy(H)[HCO_3] (**3a**) were submerged in freshly prepared unbuffered solutions of varying pH (pH 2 or pH 12) in Ace Glass pressure tubes at 25°C for 24 h. Experiments were conducted under N_2 gas to minimize the possibility of pH change due to adsorption of atmospheric CO_2. After this time, functionalized surfaces were rinsed in deionized water (3 x 2 mL) and dried in an N_2 gas stream. The pH values of the solutions were adjusted using concentrated NaOH and HCl solutions in deionized water. Unbuffered solutions were employed in order to avoid potential adsorption effects of buffer ions from solution.

THF Stability Tests

NHC films derived from *i*Pr_2bimy(H)[HCO_3] (**3a**) were placed in Ace Glass pressure tubes and 3 mL of freshly distilled THF was added. The tube was purged with nitrogen gas, sealed and heated to 68°C for 24 h. After this time, the sample was cooled to RT, rinsed in THF (2 x 5 mL), and dried under a nitrogen gas stream.

Decalin Stability Tests

NHC films derived from *i*Pr_2bimy(H)[HCO_3] (**3a**) were placed in Ace Glass pressure tubes and 2 mL of decalin were added. The tubes were purged with nitrogen gas, sealed and heated to either 100 °C or 190 ^{o}C for 24 h. After this

time, the samples were cooled to RT, rinsed with hexane (2 x 5 mL), ether (2 x 5 mL), and ethanol (2 x 5 mL), and dried under a nitrogen gas stream.

Water Stability Tests

NHC films derived from *i*Pr_2bimy(H)[HCO_3] (**3a**) were placed in freshly deionized (resistivity 18.2 MΩ·cm) water (3 mL) in Ace Glass pressure tubes at left at 25 °C or heated to 100 °C for 24 h. A separate slide was left at 25 °C for one month to assess long-term stability. Experiments were conducted under nitrogen gas. After this time, functionalized surfaces were rinsed in DI water (3 x 3 mL) and dried in a nitrogen gas stream.

Peroxide Stability Tests

NHC films derived from *i*Pr_2bimy(H)[HCO_3] (**3a**) were placed in a freshly prepared 1% solution of hydrogen peroxide and left at RT for 24 h. After this time, the surfaces were rinsed in DI water (3 x 3 mL) and dried in a nitrogen gas stream.

SPR Protocols

Preparation of Small Unilamellar Vesicles (SUVs)

SUVs were prepared in PBS buffer (100 mM Na_2HPO_4/NaH_2PO_4, 150 mM NaCl, pH 7.4). The general protocol was as follows: Egg yolk L-α-phosphatidylcholine (Sigma, P3556) (9.0 mg, 0.012 mmol) was dissolved in chloroform/methanol (2/1, v/v) in a vial. The solvent mixture was evaporated under a steam of nitrogen for at least 30 min, yielding a thin lipid film on the bottom of the vial. The lipid films were then thoroughly dried by connecting the vial to a vacuum pump for 2 h in order to remove the organic solvents. The dried lipid films were hydrated by adding 6.0 mL of the running buffer to be used in the assays (2mM). The vial was then vortexed thoroughly until all lipid films were removed from the wall of vial, resulting in a milky suspension. The lipid suspension was then frozen in a dry ice/acetone bath for 8 min, followed by thawing in a hot water bath (80 °C, 8 min). This freeze–thaw cycle was repeated 8 times. The solution was then sonicated until the suspension changed from milky to nearly transparent, yielding a uniform suspension of SUVs with a predominant size range between 30 and 35 nm.

Formation and Regeneration of Lipid Monolayer on NHC Sensor Chip

The formation and regeneration of lipid monolayer test was carried out using Biacore 3000 (GE Healthcare) and NHC sensor chips. Following equilibration of the sensor chip to room temperature, the sensor chip was docked into Biacore 3000 and primed with running buffer. All solutions for injections were prepared freshly, filtered through a 0.2 mm pore filter, and thoroughly degassed prior to use.

The sensor surface was preconditioned by a 5-min injection of 40 mM n-Octylβ-D-glucopyranoside (OG) at a flow rate of 10 mL/min. SUVs were injected immediately for a period of 25 min, followed by a 5 min dissociation period with buffer. To remove loosely bound vesicles, the flow rate was increased to 100 mL/min for a 1 min buffer rinse followed by a 1-min wash with 50 mM sodium hydroxide at 10 mL/min. The result was a stable baseline, presumably

corresponding to the lipid monolayer. The degree of the surface coverage for the lipid was evaluated by injecting 0.1 mg/mL BSA at flow rate of 10 mL/min for 5 min to assess the quantity of non-specific binding. After each binding cycle, the sensor surface was regenerated by 40 mM OG for 5 min. The stability of the sensor chip performance was assayed by repeated cycles of binding with egg PC SUV and regenerating with OG. Sensor chips were stored at 4 °C in 50 mL centrifuge tubes, which contained a small portion of moist tissue for maintaining hydration environment.
Proteins have been found to bind non-specifically to the uncoated hydrophobic surfaces, such as the HPA sensor surface. An uncoated NHC chip surface was found to bind approximately 1000 RU of bovine serum albumin (BSA), similar to the amount previously reported for HPA sensor chip surface.

We tested a range of buffers and pHs to evaluate egg PC lipid binding capacity and reproducibility over multiple binding cycles, and non-specific binding of lipid monolayer formation.[20] The buffers with pHs including (100 mM Citric acid/200 mM Na_2HPO_4) Citrate pH 5.0, (100 mM Na_2HPO_4/NaH_2PO_4, 150 mM NaCl) PBS pH 7.4, (10 mM N-(2-hydroxyethyl) 1-piperazine-N'-(2-ethanesulphonic acid), 100 mM NaCl) HEPES pH 8.0, (10 mM Tris-HCl, 1 mM EDTA) TE pH 8.0, and (10 mM 3-(Cyclohexylamino)-1-propanesulfonic acid, 150 mM NaCl) CAPS pH 10.0.

Thermal Stability of NHC Sensor Chip

Gold sensor surfaces (SIA kit Au, GE Healthcare) coated with the NHC-based SAM was baked at a constant 65ºC for 24 h, then cooled in air and mounted onto the inner support of a sensor chip (SIA kit Au, GE Healthcare). Performance of the 65ºC-exposed NHC sensor chip was evaluated by 4 cycles of lipid (egg PC, SUV) monolayer formation and regeneration in PBS buffer, as described above for the non-exposed NHC sensor chip.

Mellitin Binding Tests

Egg PC/cholesterol (10:1 w/w) was dissolved into chloroform/methanol (2/1, v/v) in a vial containing phosphate buffer (20 mM Na2HPO4/NaH2PO4, pH 6.8). SUVs for lipid/peptide test were prepared as described above with a predominant size range between 30 and 35 nm.
The lipid monolayer linked to the chip surface was then used as a model cell membrane surface to scrutinize peptide-membrane binding interactions. Melittin solutions were prepared by dissolving melittin in phosphate buffer (20 mM Na_2HPO_4/NaH_2PO_4, pH 6.8) and diluted twofold with the concentrations range from 0.3 to 0.015 µM. Then the melittin solutions were injected serially over a flow cell on the lipid surface at constant a flow rate of 10 µL/min for 4 min, and then were replaced by phosphate buffer alone to allow the melittin-lipid complex dissociate for 6 min. 10 mM NaOH was used as regeneration solution. The melittin/lipid binding data was analyzed using the same steady state affinity model as reported by Shai et al.[31] to allow for comparison.

Strepavidin

Method for preparation of NHC-SA, S-SA, and CM3-SA chip surfaces

Streptavidin was immobilized (covalently bound to the carboxymethylated dextran (CM) surface) using a standard amine-coupling procedure. PBS (10 mM

phosphate, 138 mM NaCl, 2.7 mM KCl, pH 7.4) was used as the running buffer. First, the CM type chip was activated with 0.2 M EDC/ 0.05 M NHS (EDC = 1-Ethyl-3-(3-dimethylaminopropyl)carbodiimide, NHS = N-hydroxy succinimide) for 10 min at a flow rate of 5 ul/min by modification of the carboxymethyl groups to NHS-esters. Then, 0.1 mg/ml streptavidin in 10 mM sodium acetate, pH 4.7, was injected for 10 min. Finally, the unreacted NHS-esters were deactivated by 10-min injection of 1 M ethanolamine (pH 8.5), followed by a short pulse (1 min) of 2 M NaCl at 20 ul/ml to remove any non covalently bound streptavidin and obtain a stable baseline.

The proof of concept interaction of NHC-SA, S-SA, and CM3-SA chips with D-Biotin

To provide a practical validation of the functionality of NHC-SA, S-SA, and CM3-SA chip surfaces, we have chosen to use D-biotin ($C_{10}H_{16}N_2O_3S$, MW 244.31) to prove these surfaces are applicable to use biotinylated ligands such as nucleic acids, lipids, and proteins to interact with other analytes for biosensing research.

First, 1 mM D-biotin was dissolved in PBS buffer and filtered and degassed. Then, it was injected for 5 min and sensing continued until the baseline was stable. To stabilize the baseline in a short time period, several short injections of 50 mM NaOH can be used.

Temperature Programmed Desorption (TPD) Results

TPD experiments were performed using instrumentation described in general considerations, on films vapor deposited from precursor **3a**. Assignment of the fragments is as follows: H_2 (m/z= 2), HCN (m/z= 27), C_3H_3 (m/z= 39) from the benzene moiety and C_3H_5 (m/z= 41) from the isopropyl groups. The TPD traces in [**Supplementary Figure 3**] show a main desorption peak at about T_{max}= 600 K for all the traces monitored, with some minor shifting to lower temperatures for coverages greater than ~0.5 ML. Such minor shifts can be explained by relatively weak repulsive lateral interactions of the type often observed for crowded surfaces. We interpret the desorption profile as the intact evolution of **2a** on the Au (111) surface with subsequent fragmentation at the mass spectrometer. The desorption profile is typical of first order kinetics, consistent with the proposed desorption mechanism. A simple Redhead analysis yields a desorption energy of E_{des}= 143 ±10 kJ·mol^{-1}, in very good agreement with the reported calculated bond energy of 149 kJ·mol^{-1}.[14] We have assumed a pre-exponential factor of $5x10^{11}$ s^{-1}. Note that a range of prefactors could reasonably have been used between $5x10^{10}$ and $5x10^{12}$ s^{-1}. The uncertainty in the value of the prefactor introduces the error bar of ± 10 kJ·mol^{-1} on the estimated value of the desorption energy. A peak at 510 K is also observed in some of the spectra, which we tentatively ascribe to decomposition via a minor pathway.

High Resolution Electron Energy Loss Spectroscopy (HREELS) Results

HREELS experiments were run using instrumentation described in General Considerations, on films vapor deposited from precursor **3a**. A series of HREELS spectra are given in [**Supplementary Figure 6**] as a function of preannealing temperatures after saturation exposure of **3a** on the clean Au (111) at 300 K. The assignment of peaks based on the calculated spectrum [**Supplementary Figure 7**] for a gas phase model of a NHC-Au-Cl complex of

iPr_2bimy using DFT at the SDD effective core potential/basis set on Au and TZVP basis set on all other atoms are given in [**Supplementary Table 2**]. The spectra appear consistent throughout up to 605 K where complete desorption to a clean surface is obtained, consistent with the T_{max} observed under TPD; indicating that the molecule preserves its integrity upon adsorption and heating.

The peaks observed at 2960 cm^{-1} and 3070 cm^{-1} are unequivocally assigned to C-H stretches of the isopropyl and aromatic ring respectively.[21,22] The peaks in the 1155 to 1465 cm^{-1} region correspond to a complex combination of skeletal vibrations involving C=C and C=N stretches, and C-H bending modes.[23,24]

The inhomogeneous broadening and the variations in intensity of the 500-1000 cm^{-1} region are associated with a disordered layer[25] that undergoes changes in the molecular packing with temperature. The broad loss corresponds to the coalescence of at least three peaks, mainly at 750, 870 and 930 cm^{-1}. The losses at 870 and 930 cm^{-1} are typical of skeletal vibrations of branched alkyl groups; therefore we assign these peaks to the presence of isopropyl groups. Interestingly, the energy loss arising at 750 cm^{-1} due to the ω(CH) of the benzene moiety is enhanced upon annealing. Other sets of HREELS data showed better-resolved bands in this region with similar changes for this mode. By evoking the surface selection rule[26], the observed enhancement may indicate a tilting of the ring to a more parallel orientation with respect to the surface plane.

The peak at 420 cm^{-1} is assigned to a υ(Au-C), confirming the adsorption of iPr_2bimy on the Au (111) surface via an Au–C bond. Laurentius et al. observed an energy loss also at 420 cm^{-1} for nitrobenzene films on Au (111), formed from the corresponding diazonium salt.[27] The authors report a similar band for other diazonium-derived gold nanoparticles under SERS and supported by DFT calculations. Studies on CN and CO adsorption on Au report peaks appearing between 250 and 400 cm^{-1} assigned to either stretching or bending modes of a C-Au bond.[28,29]

After annealing to 515 K, the emergence of a very weak peak at 2600 cm^{-1} is noticeable. This peak does not relate to any band in the gas phase spectrum, and has been left unassigned. We propose that its origin may be associated with a decomposition product as its appearance coincides with the small peak observed in this temperature range for some of the TPD traces. No bands are observed in the carbonyl-stretching region that could suggest the coadsorption of the carbonate counterion, in agreement with the TPD data.

STM Results for Films Prepared in Solution

Determination of pit density

Image threshold analysis [**Supplementary Figure 5**] using was performed using MATLAB to count the number and area of pits on a surface. Images were converted to a binary image where islands were set to low and all other areas were set to high. Images were chosen to contain a single atomic terrace to allow for simplified thresholding. Human inspection is used for each thresholded image to determine quality of the procedure. Adjustments to data leveling and threshold settings can be made to obtain an ideal binary image.

A histogram of pit size frequency and a mean pit size is calculated for each image. Pit density ($A_{pit}/A_{total\ image}$) for each image is also calculated. Pit density seems to collect in two ranges, one around 0.10 ± 0.05 and another around 0.35± 0.01.

Low Energy Electron Diffraction (LEED) Studies

LEED experiments were run as described in general considerations, on films vapor deposited from precursor **3a**. LEED patterns were monitored for beam energies between 0 and 120 eV and a (1x1) diffraction pattern was observed across a range of coverages and annealing temperatures indicating the absence of long-range ordered superstructures under the examined experimental conditions. The 22x√3 (herringbone) reconstruction is still visible upon adsorption of iPr_2bimy at saturation coverage as shown in [**Supplementary Figure 4**]. Molecular resolution was not attained under any tunneling condition, and streakiness was present throughout the scans, indicative of the presence of highly mobile adsorbate species. Upon heating to 500 K the etch pitting initially observed at 375 K was more pronounced; and the presence of bright features, which we believe are Au clusters, dominate the surface topography. This phenomenon resembles the behavior typically encountered in SAM of thiols on the Au substrate.[30] The extensive pitting at 500 K may be related to the additional features that appear at this temperature in the HREELS and TPD spectra.

SPR Results

The reproducibility of the NHC-derived sensor chip was benchmarked against the commercial HPA chip under a range of pH conditions. [**Supplementary Table 3**] demonstrates that the reproducibility of the phosphatocholine hybrid lipid bilayer loading was similar for both HPA and NHC under most pH conditions. However, the adsorption of BSA, which probes the quality of the surface, showed much higher variability on the HPA chip under all but the highest pH conditions, such that the NHC chip demonstrates more reproducible film quanlity. Furthermore, the absolute quantity of BSA adsorbed was higher on the HPA chip at both extremes of pH, suggesting that a more complete hybrid lipid bilayer is formed on the NHC chip over a much wider range of pH conditions.

The effect of heating the NHC-modified SPR chip is shown in [**Supplementary Table 4**] and discussed in more detail in the main text.

[Supplementary Figure 8] shows senorgrams of mellitin absorption at various concentrations on the lipid layer for both NHC and HPA chips. In each case, one observes an adsorption phase (rising signal); this is followed by a rapid decrease in signal as the adsorbed mellitin/lipid layer is removed, in preparation for the next run. On the NHC chip, the association phase approaches equilibrium

within 4 minutes, similar to what was observed by Shai *et al.*[31] for L1 chips. However, on the HPA chip each concentration produced an association response which was almost linear, which gives minimal/limited information about binding features, and we were unable to obtain reliable rate constants from this data. Unfortunately, Shai et al. did not present sensorgrams of their kinetic data from HPA chips, so this latter result cannot be directly compared to the literature. Association constants, K_A, as determined by the steady-state affinity model are summarized in [**Supplementary Table 5**]

Electrochemical Results

Films derived from NHC derivative **3c,** which has a tethered ferrocene substituent, were characterized using cyclic voltammetry at different scan rates. As shown in [**Supplementary Figure 9**], the oxidation and reduction peaks are similar sizes, although quantified charge ratio of oxidation peak to reduction peak has already demonstrated asymmetric behavior. This asymmetry is enhanced at ultra-fast scan rates, as shown in [**Supplementary Figure 9**]. The oxidation peak is very broad at a scan rate of 1000 V/s and it is almost blended into the background. On the contrary, the reduction peak holds its shape quite well, due to a faster electron transfer rate for reduction as discussed in the main text.

In order to compliment and verify the molecular density measurement shown in [**Figure 3a**], current over time was also obtained. The I vs t curves in [**Supplementary Figure 9**] have been obtained by applying constant potentials of +0.6 V and -0.2 V respectively for oxidation and reduction. Capacitive currents were subtracted by removing background current using the current level maintained after 2 mins. The width of the redox peaks shown on the CV [**Supplementary Figure 9**] measured with a scan rate of 1 V/s is approximately 0.8 V, which means the peak is obtained over a time period of 0.8 seconds. Integrating I vs t curves over similar periods of time provides a charge density of 2.6173×10^{-10} C and 2.9999×10^{-10} C for oxidation and reduction, which can be translated into a molecular density of 3.33 ± 0.10 molecules/nm^2 and 3.81 ± 0.11 molecules/nm^2. This result is consistent with values obtained using using cyclic voltammetric measurements as described in the main text.

The stability of NHC films derived from deposition of carbonate **3c**, without Fc labels, was tested under electrochemical cycling, as showing in [**Supplementary Figure 9**]. The electrochemical window was selected based on one of the common testing windows for electrochemical biosensors.[32-35] The redox signals from 5 mM/5 mM $Fe(CN)_6^{3-/4-}$ in solution were maintained at virtually the same levels after 150 cycles, with only 3% change observed. This indicates that the stability of the NHC film is high under this potential window and is as good as films obtained using the free carbene method.[14]

Supplementary References

1 Drevniok, B., Paul, W. M. P., Hairsine, K. R. & McLean, A. B. Methods and instrumentation for piezoelectric motors. *Rev Sci Instrum* **83**, 033706-6 (2012).

2 Zahl, P., Wagner, T., Moller, R. & Klust, A. Open source scanning probe microscopy control software package GXSM. *J Vac Sci Technol B* **28**, C4E39-C3E47 (2010).

3 Scofield, J. H. Hartree-Slater subshell photoionization cross-sections at 1254 and 1487 eV. *J Electron Spectrosc* **8**, 129-137 (1976).

4 Kohn, W. & Sham, L. J. Self-Consistent Equations Including Exchange and Correlation Effects. *Phys Rev* **140**, A1133-A1138 (1965).

5 Hohenberg, P. & Kohn, W. Inhomogeneous Electron Gas. *Phys Rev* **136**, B864-B871 (1964).

6 Zhao, Y. & Truhlar, D. G. The M06 suite of density functionals for main group thermochemistry, thermochemical kinetics, noncovalent interactions, excited states, and transition elements: two new functionals and systematic testing of four M06-class functionals and 12 other functionals. *Theor Chem Acc* **120**, 215-241 (2008).

7 Schafer, A., Huber, C. & Ahlrichs, R. Fully Optimized Contracted Gaussian-Basis Sets of Triple Zeta Valence Quality for Atoms Li to Kr. *J Chem Phys* **100**, 5829-5835 (1994).

8 Andrae, D., Haussermann, U., Dolg, M., Stoll, H. & Preuss, H. Energy-Adjusted Abinitio Pseudopotentials for the 2nd and 3rd Row Transition-Elements. *Theor Chim Acta* **77**, 123-141 (1990).

9 Dolg, M., Wedig, U., Stoll, H. & Preuss, H. Energy-Adjusted Abinitio Pseudopotentials for the 1st-Row Transition-Elements. *J Chem Phys* **86**, 866-872 (1987).

10 Alecu, I. M., Zheng, J. J., Zhao, Y. & Truhlar, D. G. Computational Thermochemistry: Scale Factor Databases and Scale Factors for Vibrational Frequencies Obtained from Electronic Model Chemistries. *J Chem Theory Comput* **6**, 2872-2887 (2010).

11 Chen, W. C. *et al.* Mechanistic Study of a Switch in the Regioselectivity of Hydroheteroarylation of Styrene Catalyzed by Bimetallic Ni-Al through C-H Activation. *Chem-Eur J* **20**, 8099-8105 (2014).

12 Starikova, O. V. *et al.* Synthesis of 1,3-dialkylimidazolium and 1,3-dialkylbenzimidazolium salts. *Russ J Org Chem* **39**, 1467-1470 (2003).

13 Fernandez-Rodriguez, M. A., Andina, F., Garcia-Garcia, P., Rocaboy, C. & Aguilar, E. Multicomponent Cascade Reactions Triggered by Cycloaddition of Fischer Alkoxy Alkynyl Carbene Complexes with Strained Bicyclic Olefins. *Organometallics* **28**, 361-369 (2009).

14 Crudden, C. *et al.* Ultra stable self-assembled monolayers of N-heterocyclic carbenes on gold. *Nature Chem* **6**, 553-553 (2014).

15 Kabalka, G. W., Shoup, T. M. & Goudgaon, N. M. Sodium Perborate - a Mild and Convenient Reagent for Efficiently Oxidizing Organoboranes. *J Org Chem* **54**, 5930-5933 (1989).

16 Horcas, I. *et al.* WSXM: A software for scanning probe microscopy and a tool for nanotechnology. *Rev Sci Instrum* **78**, 013705-8 (2007).

17 Barth, J. V., Brune, H., Ertl, G. & Behm, R. J. Scanning Tunneling Microscopy Observations on the Reconstructed Au(111) Surface - Atomic-Structure, Long-Range Superstructure, Rotational Domains, and Surface-Defects. *Phys Rev B* **42**, 9307-9318 (1990).

18 Granqvist, N. *et al.* Control of the Morphology of Lipid Layers by Substrate Surface Chemistry. *Langmuir* **30**, 2799-2809 (2014).

19 Lofas, S. & Johnsson, B. A Novel Hydrogel Matrix on Gold Surfaces in Surface-Plasmon Resonance Sensors for Fast and Efficient Covalent Immobilization of Ligands. *J Chem Soc Chem Comm*, 1526-1528 (1990).
20 Cooper, M. A., Try, A. C., Carroll, J., Ellar, D. J. & Williams, D. H. Surface plasmon resonance analysis at a supported lipid monolayer. *BBA-Biomembranes* **1373**, 101-111 (1998).
21 Whelan, C., Barnes, C., Walker, C. & Brown, N. Benzenethiol adsorption on Au(111) studied by synchrotron ARUPS, HREELS and XPS. *Surf Sci* **425**, 195-211 (1999).
22 Ovari, L., Wolf, M. & Tegeder, P. Reversible changes in the vibrational structure of tetra-tert-butylazobenzene on a Au(111) surface induced by light and thermal activation. *J Phys Chem-US C* **111**, 15370-15374 (2007).
23 McNutt, A., Haq, S. & Raval, R. RAIRS investigations on the orientation and intermolecular interactions of adenine on Cu(110). *Surf Sci* **531**, 131-144 (2003).
24 Prabhaharan, M., Prabakaran, A., Gunasekaran, S. & Srinivasan, S. Molecular structure and vibrational spectroscopic investigation of melamine using DFT theory calculations. *Spectrochim Acta A* **123**, 392-401 (2014).
25 Ibach, H. *Physics of Surfaces and Interfaces*. (Springer, 2006).
26 Sheppard, N. & Erkelens, J. Vibrational-Spectra of Species Adsorbed on Surfaces - Forms of Vibrations and Selection-Rules for Regular Arrays of Adsorbed Species. *Appl Spectrosc* **38**, 471-485 (1984).
27 Laurentius, L. *et al.* Diazonium-derived aryl films on gold nanoparticles: evidence for a carbon-gold covalent bond. *ACS Nano* **5**, 4219-4227 (2011).
28 Beltramo, G., Shubina, T., Mitchell, S. & Koper, M. Cyanide adsorption on gold electrodes: a combined surface enhanced Raman spectroscopy and density functional theory study. *J Electroanal Chem* **563**, 111-120 (2004).
29 Murray, C. & Bodoff, S. Cyanide adsorption on silver and gold overlayers on island films as determined by surface enhanced raman-scattering. *J Chem Phys* **85**, 573-584 (1986).
30 Poirier, G. Mechanism of formation of Au vacancy islands in alkanethiol monolayers on Au(111). *Langmuir* **13**, 2019-2026 (1997).
31 Papo, N. & Shai, Y. Exploring peptide membrane interaction using surface plasmon resonance: Differentiation between pore formation versus membrane disruption by lytic peptides. *Biochemistry-Us* **42**, 458-466 (2003).
32 Li, Y. X. *et al.* Impedance based detection of pathogenic E-coli O157:H7 using a ferrocene-antimicrobial peptide modified biosensor. *Biosens Bioelectron* **58**, 193-199 (2014).
33 Amini, K., Chan, N. W. C. & Kraatz, H. B. Toll-like receptor 3 modified Au electrodes: an investigation into the interaction of TLR3 immobilized on Au surfaces with poly(I:C). *Anal Methods-Uk* **6**, 3322-3328 (2014).
34 She, Z. *et al.* Investigation of the Utility of Complementary Electrochemical Detection Techniques to Examine the in Vitro Affinity of Bacterial Flagellins for a Toll-Like Receptor 5 Biosensor. *Anal Chem* **87**, 4218-4224 (2015).
35 Wang, N. *et al.* Clickable 5 '-gamma-Ferrocenyl Adenosine Triphosphate Bioconjugates in Kinase-Catalyzed Phosphorylations. *Chem-Eur J* **21**, 4988-4999 (2015).
